# Improved Synthesis of Asymmetric Curcuminoids and Their Assessment as Antioxidants

**DOI:** 10.3390/molecules27082547

**Published:** 2022-04-14

**Authors:** Yang-Je Cheng, Cai-Wei Li, Cing-Ling Kuo, Tzenge-Lien Shih, Jih-Jung Chen

**Affiliations:** 1 Department of Chemistry, Tamkang University, Tamsui Dist., New Taipei City 251301, Taiwan; jason0110422@gmail.com (Y.-J.C.); rose40635@gmail.com (C.-L.K.); 2 Institute of Traditional Medicine, National Yang Ming Chiao Tung University, Taipei 112304, Taiwan; leecw1219.y@nycu.edu.tw; 3 Department of Pharmacy, College of Pharmaceutical Sciences, National Yang Ming Chiao Tung University, Taipei 112304, Taiwan; 4 Department of Medical Research, China Medical University Hospital, China Medical University, Taichung 404332, Taiwan

**Keywords:** asymmetric curcuminoids, curcuminoids, Pabon’s method, 2,4-pentanedione

## Abstract

In this paper, the syntheses of twelve asymmetric curcumin analogs using Pabon’s method are reported. Generally, the previously reported yields of asymmetric curcuminoids, such as **9a** (53%), **9c** (38%), and **9k** (38%), have been moderate or low. Herein, we propose that the low yields were due to the presence of water and *n*-BuNH_2_ in the reaction media. To prove this formulated hypothesis, we have demonstrated that the yields can be improved by adding molecular sieves (MS) (4 Å) to the reaction mixture, thus reducing the interference of water. Therefore, improved yields (41–76%) were obtained, except for **9b** (36.7%), **9g** (34%), and **9l** (39.5%). Furthermore, compounds **9b**, **9d**, **9e**, **9f**, **9g**, **9h**, **9i**, **9j**, and **9l** are reported herein for the first time. The structures of these synthetic compounds were determined by spectroscopic and mass spectrometry analyses. The free radical scavenging ability of these synthetic asymmetric curcuminoids was evaluated and compared to that of the positive control butylated hydroxytoluene (BHT). Among the synthesized asymmetric curcuminoids, compounds **9a** (IC_50_ = 37.57 ± 0.89 μM) and **9e** (IC_50_ = 37.17 ± 1.76 μM) possessed effective 2,2-diphenyl-1-picrylhydrazyl (DPPH) radical scavenging abilities, and compounds **9h** (IC_50_ = 11.36 ± 0.65 μM) and **9i** (IC_50_ = 10.91 ± 0.77 μM) displayed potent 2,2’-azinobis-(3-ethylbenzthiazoline-6-sulphonate) (ABTS) radical scavenging abilities comparable to that of curcumin (IC_50_ = 10.14 ± 1.04 μM). Furthermore, all the synthetic asymmetric curcuminoids were more active than BHT.

## 1. Introduction

**Figure 1:**
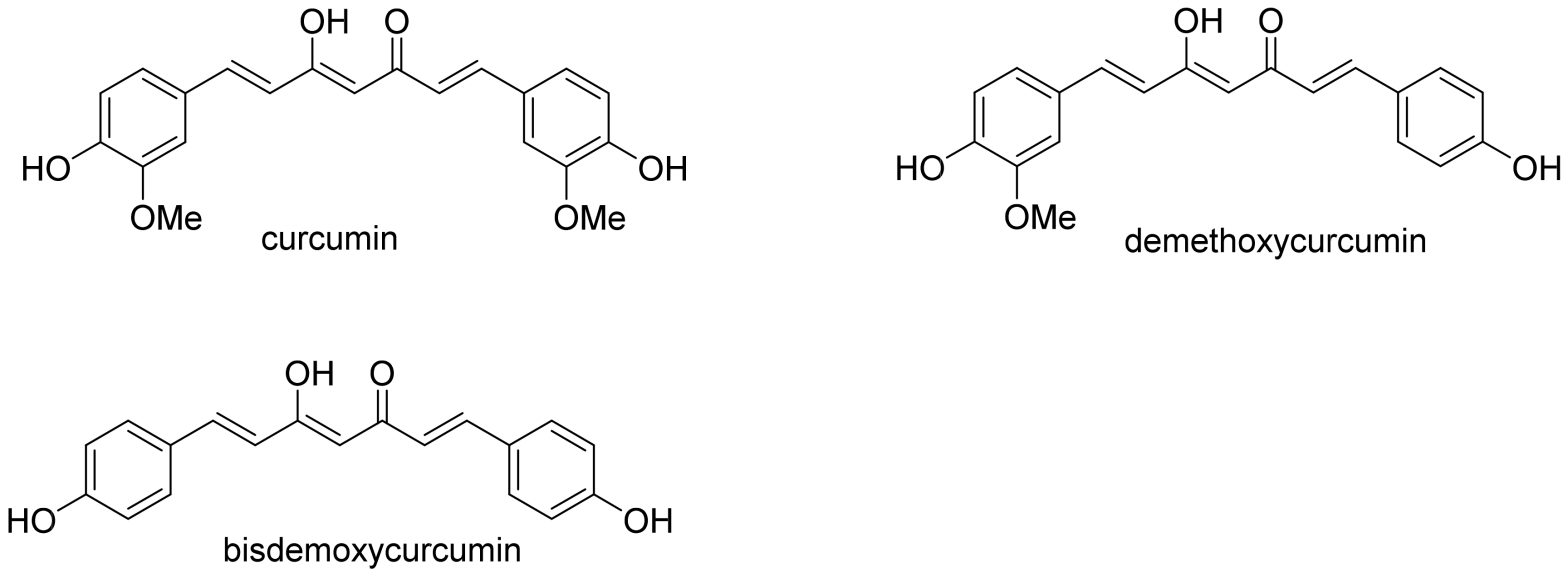
The most abundant constituents of natural curcuminoids.

**Figure 2:**
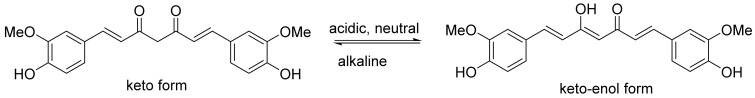
Tautomerism of curcumin under different pH values.

Turmeric is an orange-yellow powder that is obtained from the rhizomes of the natural plant *Curcuma longa*, and is used as a spice and traditional herbal medicine in South Asian countries like India and China. Although turmeric has been used for thousands of years, its major constituent curcumin was discovered in 1815 [1]. Thereafter, the structure of curcumin was determined [2], and it was synthesized [3] in the 1910s. The active components of turmeric that are used for medicinal purposes and as food additives for human health [4] are called curcuminoids. Their biological activities have attracted increasing attention from many researchers since the 1970s [5], and research on curcuminoids has grown rapidly since the 1990s [6]. The most abundant constituents of curcuminoids isolated from *Curcuma longa* are curcumin (CUR, curcumin I), demethoxycurcumin (DMC, curcumin II), and bisdemethoxycurcumin (BDMC, curcumin III), with contents of approximately 60–75%, 15–30%, and 3–15% yields, respectively (Figure 1) [7,8]. However, their contents vary depending on their origin and species [8]. Curcumin possesses a bis-α,β-unsaturated framework, which equilibrates in either a keto form or a keto-enol form in solution (Figure 2). This phenomenon is called tautomerism, which is dependent on solvent, temperature, and pH, and is determined by detailed NMR analysis [8]. Neutral or acidic conditions favor the keto-enol form, whereas the keto form is dominant under alkaline conditions [9]. Among the aforementioned curcuminoids, DMC has an asymmetric structure.

Natural curcuminoids and their synthetic derivatives exhibit a wide range of biological activities and are safe for consumers [10,11]. They possess anti-Alzheimer’s disease (AD) [12,13,14,15], anti-bacterial [16], anti-cancer [17,18,19,20,21,22,23], anti-inflammatory [12,24,25,26,27,28,29,30,31], and antioxidant properties [20,25,27,29], and are used in therapeutic treatments [9,32,33,34,35]. Curcuminoids have recently been used to synthesize diazepine derivatives, such as α-amino-3-hydroxy-5-methyl-4-isoxazole propionic acid (AMPA) receptors (AMPARs), which are involved in neurodegenerative diseases [36]. However, the limitations of using curcuminoids as pharmacological agents are due to their insolubility in water, instability, poor absorption, and rapid metabolism, which prevent their bioavailability. Furthermore, curcuminoids have been linked to over 100 cellular targets, limiting their use as therapeutic agents [12]. Therefore, improvements in the synthesis of curcuminoids are still underway, with the aim of obtaining oral bioavailable analogs [37,38].

**Figure 3:**
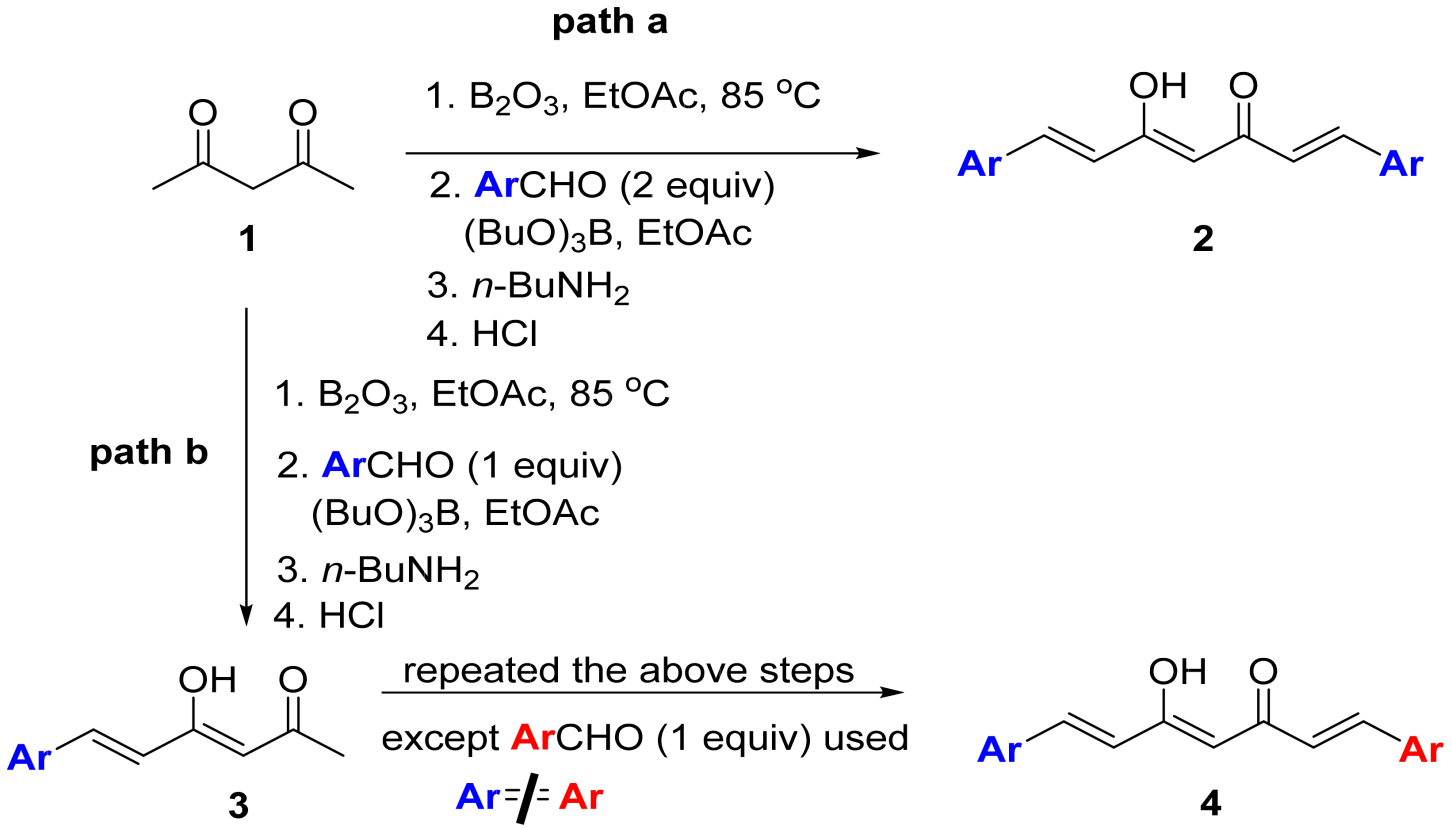
General procedure of Pabon’s method for the synthesis of symmetric and asymmetric curcuminoids.

The syntheses of symmetric or asymmetric curcumin analogs were performed using Pabon’s method [39] or modified versions of Pabon’s method [12,40,41]. Symmetric curcuminoids are generally prepared by treating 2,4-pentanedione and B_2_O_3_ in EtOAc, followed by the addition of two equivalents of the corresponding aldehydes, (BuO)_3_B and *n*-BuNH_2_, to yield **2** (Figure 3, path a). Following the same procedure, monosubstituted intermediate **3** was synthesized by adding one equivalent of aldehyde in the first step [9,12,42,43]. This intermediate can be either isolated or directly subjected to one-pot treatment with another equivalent of different aldehydes to afford **4** (Figure 3, path b). The drawback of asymmetric curcuminoid synthesis is low yields (<38–2%) [15,44]. Therefore, the synthesis of symmetric curcuminoids is more convenient, and their biological activities have been reported more routinely than those of asymmetric ones.

Oxidative stress is caused by an imbalance between free radical production and quenching. Active free radicals can react strongly with biomacromolecules, including DNA, carbohydrates, proteins, and membrane lipids, causing oxidative damage [45]. Furthermore, reactive oxygen species (ROS) play a vital role in many diseases, including atherosclerosis, cancer, mitochondrial diseases, and chronic diseases [46]. The property of hydrogen donation under oxidation allows curcumin to serve as a scavenger for most ROS [9,26,34]. Therefore, the ability to remove ROS can reduce cancer risk. In the pursuit of better oxidants than curcumin, we found that asymmetric curcumin analogs are relatively underexplored compared to symmetric ones. Therefore, the class of asymmetric curcuminoids reported here was evaluated for their antioxidant ability, and compared with curcumin and BHT as positive controls.

## 2. Results and Discussion

### 2.1. Chemistry

**Scheme 1:**
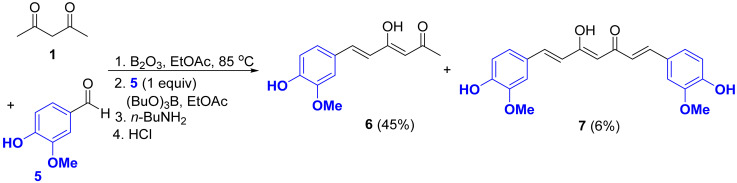
Pabon’s method for the synthesis of monosubstituted intermediate **6**.

**Scheme 2:**
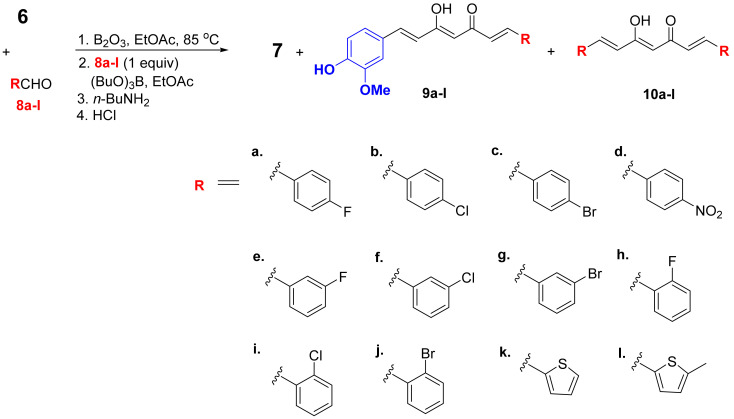
Pabon’s method for the synthesis of asymmetric curcumin analogues **9a**–**l**.

The syntheses of the asymmetric curcumin analogs are shown in Scheme 1 and Scheme 2. Monosubstituted intermediate **6** was prepared in the first step in order to synthesize asymmetric curcuminoids (Scheme 1). In this way, compound **6** was synthesized via Pabon’s method in moderate yield (45%) [22], and symmetric curcumin **7** was isolated as a by-product (6%) [16]. However, the yields of monosubstituted intermediates during the preparation of asymmetric curcuminoids have not been reported in the literature [14,15,16,21,42]. Monosubstituted **6** was therefore used as the starting material for condensation with another equivalent of the corresponding aldehydes **8a**–**l** by employing the aforementioned procedure (Scheme 2). Unfortunately, these reactions are not clean, and repeated column chromatography purification is required to afford compounds **7**, **9a**–**l**, and **10a**–**l**. The corresponding yields are listed in Table 1.

**Figure 4:**
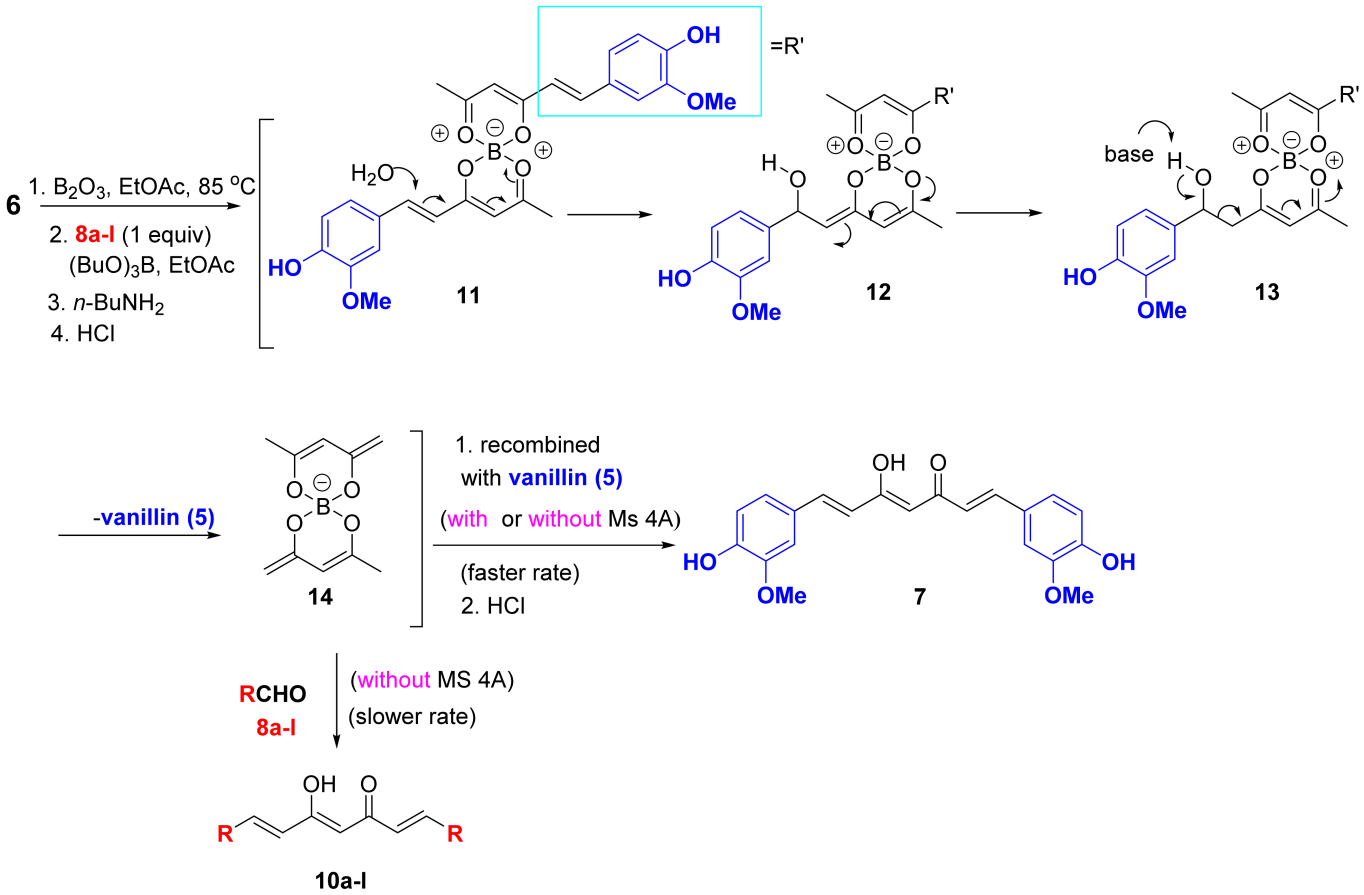
Plausible mechanism of the formation of by-products **7** and compounds **10a**–**l** for the synthesis of asymmetric curcuminoids **9a**–**l**.

The results shown in Table 1 are interesting. The combined yields of compounds **7**, **9a**–**l,** and **10a**–**l** were low to moderate (~27–59%, entries 1–12) [47]. Symmetric curcumin analogs **7** and **10a**–**l** (except **10d**) were isolated during the synthesis of asymmetric curcumin analogs. Additionally, asymmetric curcumin analogs **9a**–**l** were obtained in low yields (~11–38%) throughout this study, which is consistent with the results reported in the literature [16]. We were interested in this phenomenon and proposed a plausible mechanism for the formation of by-products **7** and **10a**–**l**, as shown in Figure 4.

First, compound **6** was complexed with B_2_O_3_ to form compound **11,** and then reacted with aldehydes **8a**–**l** in the presence of (BuO)_3_B and *n*-BuNH_2_ to afford the target compounds **9a**–**l**. Conversely, compound **11** may be hydrolyzed in the presence of trace amounts of water. Therefore, **11** underwent an accessible 1,4-addition with water to afford **12**, followed by hydrogen abstraction to afford **13**. Notably, compound **5** was recovered during this step. Compound **13** was deprotonated by *n*-BuNH_2_ via a retro aldol process to convert back to intermediate **14**, which is suspected to immediately recombine with hydrolyzed **5** to yield **7** in a faster manner [34]. Intermediate **14** also reacted with aldehydes **8a**–**l** to yield **10a**–**l** at a relatively slower rate. It has been reported that the removal of water in the reaction can increase curcumin yield [34].

The formation of intermediate **14** is most likely derived from the retro aldol reaction of boronic complex **11,** because its conjugated system serves as a Michael acceptor. Compound **11** was hydrolyzed by *n*-BuNH_2_; otherwise, compound **7** was not obtained. This evidence supports the hypothesis that the low yields of asymmetric curcuminoids are due to the presence of water. Notably, compound **10l** has previously been used to kill Gram-positive bacteria efficiently via a photodynamic method [48]. Spectroscopy data for compounds **7** and **10a**–**c,** and **10f**–**l** were compared with previously reported ones [48], except for compound **10e**.

To prove the hypothesis of the retro aldol reaction of compound **13** proposed herein, the identities of water and *n*-BuNH_2_ must be clarified. Therefore, a control experiment was designed by adding an MS of 4 Å to the reaction mixture (see Experimental Section 3.4). It was concluded that the yields of compounds **9a**–**l** were enhanced, although symmetric curcumin **7** was inevitably formed in every case (see parentheses in Table 1). However, no **10a**–**l** was isolated by column chromatography in either case in the control experiment. This proves that the removal of water from the reaction mixture favors the target molecules. It is crucial to enhance the yields of compounds **9a**–**l** and reduce the formation of compounds **10a**–**l**.

### 2.2. Pharmacology/Biology

#### 2.2.1. DPPH Free-Radical Scavenging Activity of Compounds **9a**–**l**

**Figure 5:**
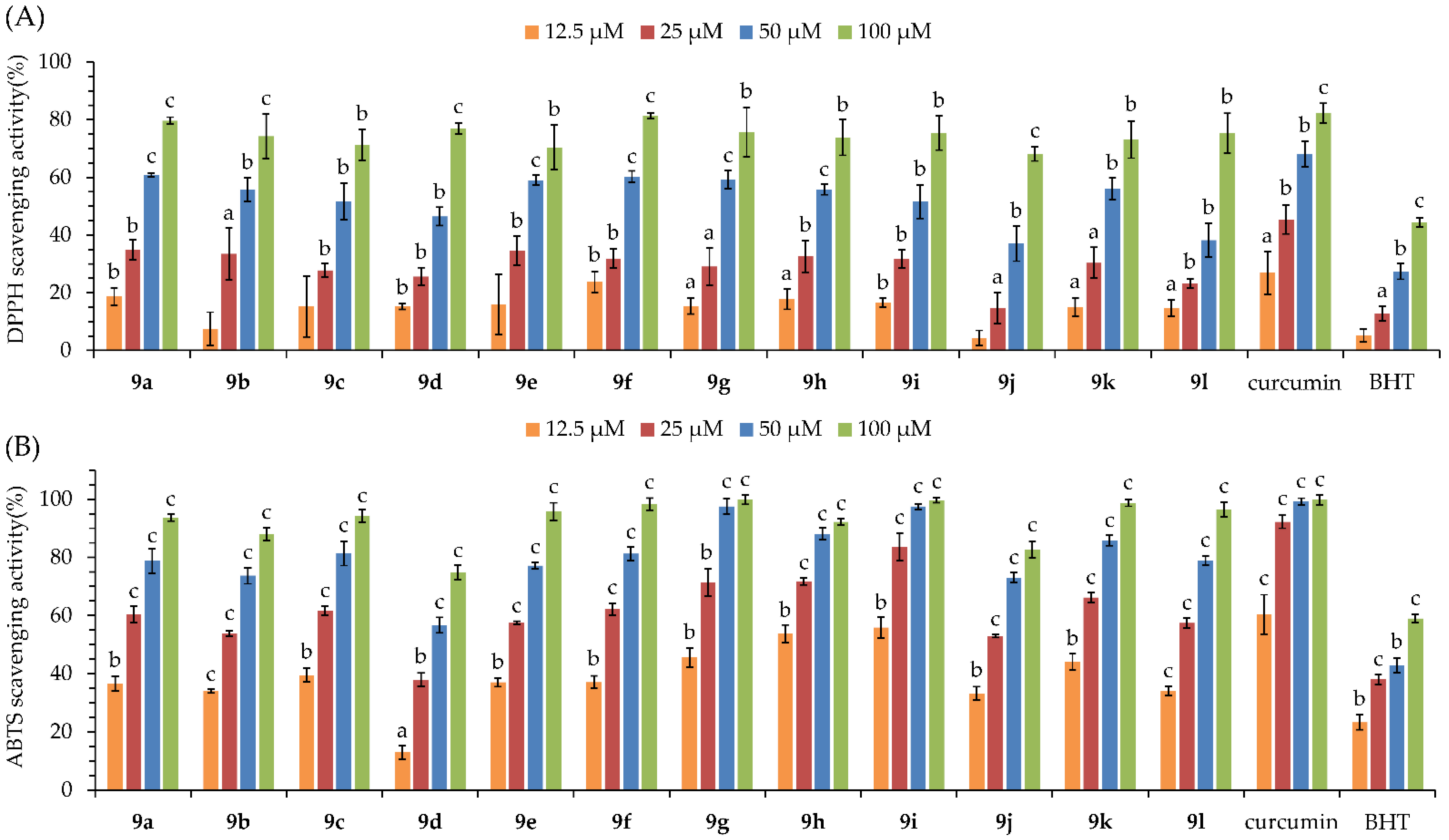
Bars show (**A**) DPPH and (**B**) ABTS radical scavenging activity at different concentrations (12.5, 25, 50, 100 μM) of compounds **9a**–**l**, curcumin, and BHT. Bars represent mean ± SD of three experiments. Bars marked with different letters are significantly different (^a^ *p* < 0.05; ^b^ *p* < 0.01; ^c^ *p* < 0.001 compared to control).

DPPH, a kind of stable free radical, accepts an electron or hydrogen radical in order to be reduced to a pale-yellow molecule [49]. The changes in absorbance can be used to determine the antioxidant capacity of the sample. The inhibitory activity data of the compounds **9a**–**l** as regards DPPH radical scavenging are shown in Figure 5 and Table 2. The commercially available antioxidant BHT was used as the positive control. From the results of the DPPH radical scavenging assay, the following conclusions can be drawn: (a) compounds **9a**–**l** exhibited potent DPPH radical scavenging activities and were more effective than the positive control, BHT (IC_50_ = 122.78 ± 0.96 μM); (b) compounds **9a** (with 4-fluorophenyl moiety) and **9e** (with 3-fluorophenyl moiety) showed the strongest DPPH radical scavenging activity among the synthesized compounds, with IC_50_ values of 37.57 ± 0.89 and 37.17 ± 1.76 μM, respectively, and displayed more effective inhibition than their analog **9h** (with 2-fluorophenyl moiety); (c) compounds **9b**, **9c**, **9f**, and **9g** (with 4-chlorophenyl, 4-bromophenyl, 3-chlorophenyl, and 3-bromophenyl moieties, respectively) exhibited more effective inhibition than their analogs, **9i** and **9j** (with 2-chlorophenyl and 2-bromophenyl moieties, respectively); (d) compound **9k** (with thiophen-2-yl moiety) displayed more effective inhibition than its analog **9l** (with 5-methylthiophen-2-yl moiety).

#### 2.2.2. ABTS Radical Cation Scavenging Activity of Compounds **9a**–**l**

Potassium persulfate converts ABTS into the blue ABTS radical cation, and it becomes colorless when neutralized by antioxidants [50]. The inhibitory activity data of the compounds **9a**–**l** in terms of ABTS radical scavenging, using BHT as the positive control, are shown in Figure 5 and Table 2. From the results of the ABTS radical scavenging assay, the following conclusions can be drawn: (a) compounds **9a**–**l** showed stronger ABTS radical scavenging activities than BHT (IC_50_ = 71.94 ± 1.81 μM); (b) compound **9i** proved the most effective among the synthesized compounds, with IC_50_ = 10.91 ± 0.77 μM, in the scavenging of the ABTS free radicals; (c) compounds **9h** and **9i** (with 2-fluorophenyl and 2-chlorophenyl moieties, respectively) displayed more effective inhibition than their analogs **9a**, **9b**, **9e**, and **9f** (with 4-fluorophenyl, 4-chlorophenyl, 3-fluorophenyl, and 3-chlorophenyl moieties, respectively); (d) compound **9g** (with 3-bromophenyl moiety) showed stronger ABTS inhibitory action than its analogs **9c** and **9j** (with 4-bromophenyl and 2-bromophenyl moieties, respectively); (e) compound **9k** (with thiophen-2-yl moiety) exhibited more effective inhibition than its analog **9l** (with 5-methylthiophen-2-yl moiety).

## 3. Materials and Methods

### 3.1. General Procedures

All chemicals were purchased from the Uni-Onward Corporation in Taiwan and used without further purification. Ethyl acetate was dried over CaH_2_ and distilled before use. Flash column chromatography was performed on 230–400 mesh SiO_2_ gel (SiliaFlash^®^ P60, 40–63 μm 60 Å; SiliCycle^®^ Inc., Quebec City, QC, Canada). Structural determinations were based on ^1^H and ^13^C nuclear magnetic resonance spectroscopy (NMR) data and were recorded on a Bruker 600 MHz Ultrashield instrument. The chemical shifts were reported in parts per million (ppm) relative to the residual of the solvents: ^1^H (7.26 ppm) and ^13^C (77.0 ppm) for CDCl_3_, and ^1^H (4.78 ppm) and ^13^C (49.15 ppm) for CD_3_OD. Melting points were measured using an MP-2D apparatus and were uncorrected. The molecular weights of the compounds were determined by high-resolution mass spectrometry (HRMS) using a TMS-700 instrument in the electrospray ionization (ESI) mode. MS 4 Å was oven-dried overnight at 100 °C before use.

### 3.2. Synthesis of Monosubstituted ***6*** (Pabon’s Method)

An oven-dried flask was charged with a mixture of 2,4-pentanedione (1.09 mL, 10.60 mmol) and B_2_O_3_ (0.5130 g, 7.33 mmol) in dry EtOAc (4.0 mL), and then heated at 85 °C for 30 min. A mixture of compound **5** (0.807 g, 5.30 mmol) and (BuO)_3_B (0.372 mL, 1.38 mmol) in EtOAc (3.0 mL) was stirred at ambient temperature for 30 min before this mixture was added to the aforementioned solution. This mixture was heated at 85 °C for 30 min, and *n*-BuNH_2_ (0.21 mL, 2.12 mmol) and EtOAc (1.0 mL) were added to the mixture using a syringe pump at 0.5 mL/h. The reaction was monitored by TLC till completion (18–24 h, depending on the aldehydes used; the reaction time for aldehyde **8d** was 48 h). The mixture was then cooled to 50 °C, followed by the addition of 2 N HCl (5.5 mL) and stirring for 30 min. Then the mixture was diluted in H_2_O and extracted with EtOAc. The organic layer was dried (MgSO_4_), concentrated, and purified using flash column chromatography.

### 3.3. Synthesis of Asymmetric Curcumin Analogs

Compound **6** (1 equivalent) was reacted with B_2_O_3_ (0.5 equivalent), (BuO)_3_B (2 equivalent), and *n*-BuNH_2_ (1 equivalent) by Pabon’s method, except that different aldehydes (2 equivalents) were used. The organic layer was purified by flash column chromatography to obtain compounds **7**, **9a**–**l**, and **10a**–**l**.

### 3.4. Controlled Experiment

A mixture of compound **6** (1 equivalent), B_2_O_3_ (0.5 equivalent), and MS 4 Å (3.5-fold *w*/*w* relative to the amount of compound **6**) in dry EtOAc was heated at 85 °C for 30 min. We then followed the procedure described in Section 3.2. The crude mixture was purified using column chromatography. Please note that using more than 3.5 times the amount of MS 4 Å gave rise to low yields of compounds **9a**–**l**.

### 3.5. Biological Assay

#### 3.5.1. Materials

2,2-Diphenyl-1-picrylhydrazyl (DPPH) was obtained from the Tokyo Chemical Industry Co., Ltd. (Tokyo, Japan). 2,2’-Azino-bis(3-ethylbenzothiazoline-6-sulfonic acid) (ABTS) was purchased from Sigma-Aldrich (St. Louis, MO, USA). Potassium peroxodisulfate was obtained from SHOWA Chemical Co., Ltd. (Chuo-ku, Japan).

#### 3.5.2. DPPH Radical Scavenging Assay

The DPPH radical-scavenging activity of each compound was measured as previously described [51]. Briefly, the DPPH solution (400 µM, dissolved in ethanol, 100 µL) and different concentrations (6.25, 12.5, 25, 50, 100, and 200 μM) of each compound (dissolved in ethanol, 100 µL) were mixed in a 96-well microplate. After incubation in the dark at room temperature for 30 min, the absorbance was measured at 520 nm using an ELISA plate reader (µ Quant; Tecan Group Ltd., Switzerland) (µ Quant). The DPPH radical scavenging activity was determined using the following formula:

DPPH radical inhibiting activity (%) = (Ac − At)/Ac × 100,

Here, Ac and At are the absorbances of the control (untreated group) and test sample, respectively. Commercially available BHT was used as a positive control. All half-maximal inhibitory concentration (IC_50_) values of the tested activities were determined using linear regression of the percentage of remaining radicals against the sample concentration.

#### 3.5.3. ABTS Cation Radical Scavenging Activity

The ABTS cation radical scavenging activity was determined using the reference method [50]. The mixture of 28 mM ABTS solution and 9.6 mM potassium peroxydisulfate (1/1, *v*/*v*) was incubated in the dark at room temperature for 16 h to prepare the ABTS cation solution. The solution was diluted with ethanol to obtain an absorbance of 0.70 ± 0.02 at 740 nm. Different concentrations of extracts (10 μL) were then mixed with the working solution (190 μL) and incubated at room temperature for 6 min. ABTS cation radical scavenging activity was determined by calculating the decrease in absorbance measured at 740 nm using the following equation:

ABTS radical inhibiting activity (%) = (Ac − At)/Ac × 100,

Commercially available BHT was used as a positive control. All the IC_50_ values of the tested activities were determined using linear regression of the percentage of remaining radicals against the sample concentration.

#### 3.5.4. Statistical Analysis

All data are expressed as mean ± standard deviation (SD). Statistical analysis was performed using Student’s *t*-test. A probability of ≤ 0.05 or less was considered statistically significant. All experiments were performed at least three times.

### 3.6. Synthetic Compounds

The following reactions were all conducted under the Section 3.4.

#### 3.6.1. (E)-6-(4-Hydroxy-3-methoxyphenyl)hex-5-ene-2,4-dione (**6**)

Purification by flash chromatography (EtOAc:Hexane = 1:4–1:2–1:1; EtOAc:Hexane = 1:2, R*_f_* = 0.6) afforded a yellow solid. Yield: 45%. (*literature* [22] 45%; [23] 50%). Mp147–149 °C (*literature* [22,23] 146–147 °C). ^1^H NMR (600 MHz, CDCl_3_) *δ* 7.53 (d, *J* = 15.8 Hz, 1H), 7.08 (dd, *J* = 8.2, 1.7 Hz, 1H), 7.00 (d, *J* = 1.7 Hz, 1H), 6.92 (d, *J* = 8.2 Hz, 1H), 6.32 (d, *J* = 15.8 Hz, 1H), 3.93 (s, 3H), 2.17 (s, 3H). ^13^C NMR (150 MHz, CDCl_3_) *δ* 196.9, 177.9, 147.7, 146.8, 140.0, 127.7, 122.6, 120.3, 114.8, 109.5, 100.7, 55.9, 26.8. HRMS (ESI) calculated for C_13_H_13_O_4_ [M–H]^+^ 233.0814. Found: 233.0817.

#### 3.6.2. (1E,6E)-1-(4-Fluorophenyl)-7-(4-hydroxy-3-methoxyphenyl)hepta-1,6-diene-3,5-dione (**9a**)

Purification by flash chromatography (EtOAc:Hexane = 1:3–1:1; EtOAc:Hexane = 1:2, R*_f_* = 0.3) afforded an orange-red solid. Yield: 58% (*literature* [16] 53%). Mp 154–155 °C (*literature* [16] 146–147 °C). ^1^H NMR (600 MHz, CDCl_3_) *δ* 7.60 (d, *J* = 15.7 Hz, 2H), 7.53 (dd, *J* = 8.6, 5.4 Hz, 2H), 7.13 (dd, *J* = 8.2, 1.6 Hz, 1H), 7.08 (t, *J* = 8.5 Hz, 2H), 7.05 (d, *J* = 1.6 Hz, 1H), 6.93 (d, *J* = 8.0 Hz, 1H), 6.59 (d, *J* = 15.8 Hz, 1H), 6.53 (d, *J* = 15.8 Hz, 1H), 5.89 (s, 1H), 5.81 (s, 1H), 3.95 (s, 3H). ^13^C NMR (150 MHz, CDCl_3_) *δ* 184.3, 182.1, 163.7 (^1^*J*_C-F_ = 249.0 Hz), 148.0, 146.8, 141.1, 138.8, 131.3, 129.8 (^3^*J*_C-F_ = 7.5 Hz), 127.6, 123.8, 123.0, 121.7, 115.9 (^2^*J*_C-F_ = 22.5 Hz), 114.8, 109.6, 101.5, 56.0. HRMS (ESI) calculated for C_20_H_18_FO_4_ [M+H]^+^ 341.1189. Found: 341.1184.

#### 3.6.3. (1E,6E)-1-(4-Chlorophenyl)-7-(4-hydroxy-3-methoxyphenyl)hepta-1,6-diene-3,5-dione (**9b**)

Purification by flash chromatography (EtOAc:Hexane = 1:3–1:1; EtOAc:Hexane = 1:2, R*_f_* = 0.4) afforded a red solid. Yield: 36.7%. Mp 115–120 °C. ^1^H NMR (600 MHz, CDCl_3_) *δ* 7.61 (d, *J* = 15.4 Hz, 1H), 7.58 (d, *J* = 15.4 Hz, 1H), 7.47 (d, *J* = 8.5 Hz, 2H), 7.36 (d, *J* = 8.5 Hz, 2H), 7.12 (dd, *J* = 8.2, 1.7 Hz, 1H), 7.05 (d, *J* = 1.7 Hz, 1H), 6.93 (d, *J* = 8.2 Hz, 1H), 6.56 (d, *J* = 15.8 Hz, 1H), 6.49 (d, *J* = 15.8 Hz, 1H), 5.92 (s, 1H), 5.81 (s, 1H), 3.94 (s, 3H). ^13^C NMR (150 MHz, CDCl_3_) *δ* 184.7, 181.5, 148.0, 146.8, 141.3, 138.6, 135.8, 133.6, 129.2, 127.5, 124.5, 123.1, 121.7, 114.9, 109.7, 101.7, 56.0. HRMS (ESI) calculated for C_20_H_18_ClO_4_ [M+H]^+^ 357.0894. Found: 357.0905.

#### 3.6.4. (1E,6E)-1-(4-Bromophenyl)-7-(4-hydroxy-3-methoxyphenyl)hepta-1,6-diene-3,5-dione (**9c**)

Purification by flash chromatography (EtOAc:Hexane = 1:3–1:1; EtOAc:Hexane = 1:2, R*_f_* = 0.46) afforded an orange solid. Yield: 73% (*literature* [16] 32%; [44] 38%). Mp 113–116 °C (*literature* [16] 148–149 °C). ^1^H NMR (600 MHz, CDCl_3_) *δ* 7.61 (d, *J* = 15.8 Hz, 1H), 7.57 (d, *J* = 15.8 Hz, 1H), 7.52 (d, *J* = 8.3 HZ, 2H), 7.41 (d, *J* = 8.3 Hz, 2H), 7.13 (dd, *J* = 8.2, 1.4 Hz, 1H), 7.05 (d, *J* = 1.3 Hz, 1H), 6.94 (d, *J* = 8.2 Hz, 1H), 6.58 (d, *J* = 15.8 Hz, 1H), 6.49 (d, *J* = 15.8 Hz, 1H), 5.89 (s, 1H), 5.81 (s, 1H), 3.93 (s, 3H). ^13^C NMR (150 MHz, CDCl_3_) *δ* 184.8, 181.4, 148.1, 146.8, 141.3, 138.6, 134.0, 132.1, 129.4, 127.5, 124.6, 124.1, 123.1, 121.8, 114.9, 109.7, 101.7, 56.0. HRMS (ESI) calculated for C_20_H_18_^79^BrO_4_ [M+H]^+^ 401.0383. Found: 401.0383.

#### 3.6.5. (1E,6E)-1-(4-Hydroxy-3-methoxyphenyl)-7-(4-nitrophenyl)hepta-1,6-diene-3,5-dione (**9d**)

Purification by flash chromatography (EtOAc:Hexane = 1:3–1:1; EtOAc:Hexane = 1:2, R*_f_* = 0.45) afforded an orange-red solid. Yield: 20% (27% based on the recovery of compound **6**). Mp 149–151 °C. ^1^H NMR (600 MHz, CDCl_3_) *δ* 8.25 (d, *J* = 8.8, 2H), 7.68 (d, *J* = 8.8 Hz, 2H), 7.67 (d, *J* = 15.8 Hz, 1H), 7.65 (d, *J* = 15.8 Hz, 1H), 7.15 (dd, *J* = 8.3, 1.9 Hz, 1H), 7.07 (d, *J* = 1.9 Hz, 1H), 6.95 (d, *J* = 8.2 Hz, 1H), 6.71 (d, *J* = 15.8 Hz, 1H), 6.53 (d, *J* = 15.8 Hz, 1H), 5.88 (s, 1H), 5.87 (s, 1H), 3.96 (s, 3H). ^13^C NMR (150 MHz, CDCl_3_) *δ* 186.4, 179.0, 148.3, 148.1, 146.9, 142.2, 141.4, 136.7, 128.4 (2 X), 127.9, 127.4, 124.2 (2 X), 123.3, 121.8, 114.9, 109.7, 102.4, 56.0. HRMS (ESI) calculated for C_20_H_16_NO_6_ [M–H]^+^ 366.0978. Found: 366.0975.

#### 3.6.6. (1E,6E)-1-(3-Fluorophenyl)-7-(4-hydroxy-3-methoxyphenyl)hepta-1,6-diene-3,5-dione (**9e**)

Purification by flash chromatography (EtOAc:Hexane = 1:3–1:1; EtOAc:Hexane = 1:2, R*_f_* = 0.5) afforded an orange solid. Yield: 51.6%. Mp 154–155 °C. ^1^H NMR (600 MHz, CD_3_OD) *δ* 7.58 (d, *J* = 15.8 Hz, 1H), 7.54 (d, *J* = 15.8 Hz, 1H), 7.40–7.37 (m, 1H), 7.34 (d, *J* = 8.0, 1.8 Hz, 1H), 7.18 (d, *J* = 1.8 Hz, 1H), 7.10–7.06 (m, 2H), 6.81 (d, *J* = 8.2 Hz, 1H), 6.76 (d, *J* = 15.8 Hz, 1H), 6.61 (d, *J* = 15.8 Hz, 1H), 5.99 (s, 1H), 3.89 (s, 3H). ^13^C NMR (150 MHz, CD_3_OD) *δ* 187.2, 181.8, 164.6 (^1^*J*_C-F_ = 243.0 Hz), 150.7, 149.4, 143.2, 139.5, 139.1 (^3^*J*_C-F_ = 7.5 Hz), 131.7 (^3^*J*_C-F_ = 7.5 Hz), 128.4, 126.5, 125.3, 124.4, 122.4, 117.5 (^2^*J*_C-F_ = 21.0 Hz), 116.6, 115.0 (^2^*J*_C-F_ = 23.0 Hz), 111.9, 102.7, 56.5. HRMS (ESI) calculated for C_20_H_18_FO_4_ [M+H]^+^ 341.1189. Found: 341.1189.

#### 3.6.7. (1E,6E)-1-(3-Chlorophenyl)-7-(4-hydroxy-3-methoxyphenyl)hepta-1,6-diene-3,5-dione (**9f**)

Purification by flash chromatography (EtOAc:Hexane = 1:3–1:1; EtOAc:Hexane = 1:2, R*_f_* = 0.4) afforded an orange-red solid. Yield: 49.2%. Mp 67–71 °C. ^1^H NMR (600 MHz, CD_3_OD) *δ* 7.64 (s, 1H), 7.61 (d, *J* = 15.8 Hz, 1H), 7.55 (d, *J* = 16.1 Hz, 1H), 7.53 (d, *J* = 9.0 Hz, 1H), 7.40–7.35 (m, 2H), 7.21 (d, *J* = 1.5 Hz, 1H), 7.11 (dd, *J* = 8.4, 1.8 Hz, 1H), 6.82 (d, *J* = 7.8 Hz, 1H), 6.80 (d, *J* = 13.2 Hz, 1H), 6.65 (d, *J* = 15.8 Hz, 1H), 6.02 (s, 1H), 3.91 (s, 3H). ^13^C NMR (150 MHz, CD_3_OD) *δ* 187.3, 181.7, 150.8, 149.5, 143.2, 139.1, 138.8, 136.0, 131.5, 130.7, 128.7, 128.4, 127.5, 126.7, 124.0, 122.4, 116.6, 111.4, 102.7. HRMS (ESI) calculated for C_20_H_18_ClO_4_ [M+H]^+^ 357.0894. Found: 357.0893.

#### 3.6.8. (1E,6E)-1-(3-Bromophenyl)-7-(4-hydroxy-3-methoxyphenyl)hepta-1,6-diene-3,5-dione (**9g**)

Purification by flash chromatography (EtOAc:Hexane = 1:3–1:1; EtOAc:Hexane = 1:2, R*_f_* = 0.5) afforded an orange solid. Yield: 34%. Mp 74–78 °C. ^1^H NMR (600 MHz, CDCl_3_) *δ* 7.70 (s, 1H), 7.61 (d, *J* = 15.8 Hz, 1H), 7.55 (d, *J* = 15.8 Hz, 1H), 7.49 (d, *J* = 7.9 Hz, 1H), 7.45 (d, *J* = 7.7 Hz, 1H), 7.26 (t, *J* = 7.6 Hz, 1H), 7.13 (dd, *J* = 8.2, 1.8 Hz, 1H), 7.06 (d, *J* = 1.7 Hz, 1H), 6.94 (d, *J* = 8.2 Hz, 1H), 6.58 (d, *J* = 15.8 Hz, 1H), 6.50 (d, *J* = 15.8 Hz, 1H), 5.89 (s, 1H), 5.82 (s, 1H), 3.95 (s, 3H). ^13^C NMR (150 MHz, CDCl_3_) *δ* 185.2, 180.9, 148.1, 146.8, 141.5, 138.2, 137.3, 132.6, 130.5, 130.4, 127.5, 126.8, 125.3, 123.2, 123.0, 121.8, 114.9, 109.7, 101.8, 56.0. HRMS (ESI) calculated for C_20_H_18_^79^BrO_4_ [M+H]^+^ 401.0388. Found: 401.0381.

#### 3.6.9. (1E,6E)-1-(2-Fluorophenyl)-7-(4-hydroxy-3-methoxyphenyl)hepta-1,6-diene-3,5-dione (**9h**)

Purification by flash chromatography (EtOAc:Hexane = 1:3–1:1; EtOAc:Hexane = 1:2, R*_f_* = 0.5) afforded an orange-yellow solid. Yield: 44.7%. Mp 124–127 °C. ^1^H NMR (600 MHz, CDCl_3_) *δ* 7.75 (d, *J* = 16.0 Hz, 1H), 7.61 (d, *J* = 15.8 Hz, 1H), 7.55 (td, *J* = 7.6, 1.4 Hz, 1H), 7.36–7.31 (m, 1H), 7.16 (t, *J* = 7.6 Hz, 1H), 7.12 (dd, *J* = 8.2, 1.9 Hz, 1H), 7.10 (dd, *J* = 10.1, 8.4 Hz, 1H), 7.05 (d, *J* = 1.8 Hz, 1H), 6.93 (d, *J* = 8.2 Hz, 1H), 6.72 (d, *J* = 16.1 Hz, 1H), 6.50 (d, *J* = 15.8 Hz, 1H), 5.91 (s, 1H), 5.85 (s, 1H), 3.95 (s, 3H). ^13^C NMR (150 MHz, CDCl_3_) *δ* 184.9, 181.5, 161.4 (^1^*J*_C-F_ = 253.5 Hz), 148.0, 146.8, 141.2, 132.7, 131.2 (^3^*J*_C-F_ = 7.5 Hz), 129.2, 127.6, 126.6 (^3^*J*_C-F_ = 6.0 Hz), 124.5, 123.2, 123.1, 121.8, 116.2 (^2^*J*_C-F_ = 21.0 Hz), 114.8, 109.2, 101.7, 56.0. HRMS (ESI) calculated for C_20_H_18_FO_4_ [M+H]^+^ 341.1189. Found: 341.1189.

#### 3.6.10. (1E,6E)-1-(2-Chlorophenyl)-7-(4-hydroxy-3-methoxyphenyl)hepta-1,6-diene-3,5-dione (**9i**)

Purification by flash chromatography (EtOAc:Hexane = 1:3–1:1; EtOAc:Hexane = 1:2, R*_f_* = 0.33) afforded an orange-red solid. Yield: 52.1%. Mp 118–120 °C. ^1^H NMR (600 MHz, CDCl_3_) *δ* 8.30 (d, *J* = 15.8 Hz, 1H), 7.66–7.63 (m, 1H), 7.61 (d, *J* = 15.8 Hz, 1H), 7.43–7.40 (m, 1H), 7.30–7.26 (m, 2H), 7.12 (dd, *J* = 8.3, 1.3 Hz, 1H), 7.05 (s, 1H), 6.93 (d, *J* = 8.2 Hz, 1H), 6.59 (d, *J* = 15.8 Hz, 1H), 6.50 (d, *J* = 15.8 Hz, 1H), 5.95 (s, 1H), 5.86 (s, 1H), 3.93 (s, 3H). ^13^C NMR (150 MHz, CDCl_3_) *δ* 184.9, 181.3, 148.0, 146.8, 141.3, 135.7, 135.0, 133.3, 130.7, 130.2, 127.5, 127.0, 126.5, 123.1, 121.8, 116.2, 114.8, 109.6, 101.5, 55.9 HRMS (ESI) calculated for C_20_H_18_ClO_4_ [M+H]^+^ 357.0894. Found: 357.0891.

#### 3.6.11. (1E,6E)-1-(2-Bromophenyl)-7-(4-hydroxy-3-methoxyphenyl)hepta-1,6-diene-3,5-dione (**9j**)

Purification by flash chromatography (EtOAc:Hexane = 1:3–1:1; EtOAc:Hexane = 1:2, R*_f_* = 0.4) afforded an orange solid. Yield: 40.9%. Mp 132–133 °C. ^1^H NMR (600 MHz, CD_3_OD) *δ* 7.93 (d, *J* = 15.8 Hz, 1H), 7.77 (d, *J* = 6.9, 1.0 Hz, 1H), 7.62 (dd, *J* = 7.6, 0.8 Hz, 1H), 7.60 (d, *J* = 15.9 Hz, 1H), 7.37 (t, *J* = 7.5 Hz, 1H), 7.24 (td, *J* = 8.6, 1.4 Hz, 1H), 7.20 (d, *J* = 1.6 Hz, 1H), 7.10 (dd, *J* = 8.2, 1.7 Hz, 1H), 6.81 (d, *J* = 8.2 Hz, 1H), 6.73 (d, *J* = 15.8 Hz, 1H), 6.64 (d, *J* = 15.8 Hz, 1H), 4.67 (s, 1H), 3.90 (s, 3H). ^13^C NMR (150 MHz, CD*_3_*OD)*δ* 187.8, 181.3, 150.9, 149.6, 143.5, 138.8, 136.4, 134.7, 132.3, 129.3, 129.0, 128.5, 127.9, 126.4, 124.6, 122.6, 116.8, 112.0, 56.6. HRMS (ESI) calculated for C_20_H_18_^79^BrO_4_ [M+H]^+^ 401.0388. Found: 401.0403.

#### 3.6.12. (1E,6E)-1-(4-Hydroxy-3-methoxyphenyl)-7-(thiophen-2-yl)hepta-1,6-diene-3,5-dione (**9k**) 

Purification by flash chromatography (EtOAc:Hexane = 1:3–1:1; EtOAc:Hexane = 1:2, R*_f_* = 0.31) afforded a red solid. Yield: 48.6% (*literature* [23] 38%). Mp 127–129 °C (*literature* [23] Mp 130–132 °C). ^1^H NMR (600 MHz, CDCl_3_) *δ* 7.76 (d, J = 15.5 Hz, 1H), 7.59 (d, *J* = 15.8 Hz, 1H), 7.38 (d, *J* = 5.1 Hz, 1H), 7.25 (s, 2H), 7.12 (dd, *J* = 8.2, 1.8 Hz, 1H), 7.06 (d, *J* = 8.6 Hz, 1H), 7.05 (dd, *J* = 8.2, 1.9 Hz, 1H), 6.93 (d, *J* = 8.2 Hz, 1H), 6.47 (d, *J* = 15.8 Hz, 1H), 6.41 (d, *J* = 15.5 Hz, 1H), 5.89 (s, 1H), 5.77 (s, 1H), 3.95 (s, 3H). ^13^C NMR (150 MHz, CDCl_3_) *δ* 183.9, 182.0, 147.9, 146.8, 140.9, 140.6, 132.8, 130.7, 128.2 (2 X), 127.6, 123.1, 123.0, 121.8, 114.8, 109.6, 101.5, 56.0. HRMS (ESI) calculated for C_18_H_17_O_4_S [M+H]^+^ 329.0848. Found: 329.0851.

#### 3.6.13. (1E,6E)-1-(4-Hydroxy-3-methoxyphenyl)-7-(5-methylthiophen-2-yl)hepta-1,6-diene-3,5-dione (**9l**)

Purification by flash chromatography (EtOAc:Hexane = 1:3–1:1; EtOAc:Hexane = 1:2, R*_f_* = 0.35) afforded an orange-red solid. Yield: 39.5%. Mp 155–157 °C. ^1^H NMR (600 MHz, CDCl_3_) *δ* 7.68 (d, *J* = 15.4 Hz, 1H), 7.57 (d, *J* = 15.7 Hz, 1H), 7.11 (dd, *J* = 8.2, 1.6 Hz, 1H), 7.06 (d, *J* = 3.5 Hz, 1H), 7.04 (d, *J* = 1.6 Hz, 1H), 6.93 (d, *J* = 8.2 Hz, 1H), 6.71 (d, *J* = 3.3 Hz, 1H), 6.46 (d, *J* = 15.7 Hz, 1H), 6.27 (d, *J* = 15.4 Hz, 1H), 5.74 (s, 1H), 3.93 (s, 3H), 2.51 (s, 3H). ^13^C NMR (150 MHz, CDCl_3_) *δ* 183.3, 182.7, 147.9, 146.8, 144.0, 140.5, 138.6, 133.4, 131.6, 127.7, 126.7, 122.9, 121.8, 114.8, 109.6, 101.3, 56.0, 15.9. HRMS (ESI) calculated for C_19_H_19_O_4_S [M+H]^+^ 343.1004. Found: 343.1010.

## 4. Conclusions

All conditions used for the synthesis of asymmetric curcumin analogues were similar, and their yields reported in the literature were low to moderate. Our results clearly indicate that the moderate yield of monosubstituted **6** is due to the competitive formation of bissubstituted **7**. The subsequent step also involves the cleavage of **11** by water, mediated by *n*-BuNH_2_, to yield three different compounds, as shown in Figure 3. Our finding is crucial and provides the optimized conditions for the synthesis of asymmetric curcuminoids by adding MS 4Å. In conclusion, compound **9a**–**l** possessed antioxidant potential and might be useful against free radical-induced disorders. It is worthy of note that symmetric **10l** was previously investigated for its photodynamic killing of Gram-positive microbes under blue light, with great potential. Compounds **9a**–**l** will be evaluated via their photodynamic effects on bacteria, and the results will be reported in due course. The synthetic compounds **9a**–**l** showed more potent DPPH and ABTS radical scavenging activities than the known antioxidant BHT. The present study suggests that the bioactive synthetic compounds **9a**–**l** (especially **9a**, **9e**, **9h** and **9i**) are worthy of further investigation, and should developed as candidates for the treatment or prevention of oxidative stress-related diseases.

## Figures and Tables

**Table 1:** Yields of products for the synthesis of asymmetric curcumin analogues **9a**–**l** from compound **6** in condensation with aldehydes **8a**–**l** in Scheme 2.

Entry	Aldehyde	Compound	Yield%	Compound	Yield%	Compound	Yield%
1	**8a**	**7**	12 (9.2 ^a^)	**9a**	28 (58 ^a^)	**10a**	2 (NA ^a^)
2	**8b**	**7**	8 (9.5 ^a^)	**9b**	27 (36.7 ^a^)	**10b**	3 (NA ^a^)
3	**8c**	**7**	7 (12 ^a^)	**9c**	22 (73 ^a^)	**10c**	3 (NA ^a^)
4	**8d**	**7**	14.3 (3.8 ^a^)	**9d**	12.5 (20 ^a^)	**10d**	NA (NA ^a^)
5	**8e**	**7**	8.4 (6 ^a^)	**9e**	28.9 (51.6 ^a^)	**10e**	3 (NA ^a^)
6	**8f**	**7**	10 (12.7 ^a^)	**9f**	34.8 (49.2 ^a^)	**10f**	4 (NA ^a^)
7	**8g**	**7**	9.4 (2.5 ^a^)	**9g**	31.1 (34 ^a^)	**10g**	9 (NA ^a^)
8	**8h**	**7**	10.6 (3.1 ^a^)	**9h**	36.8 (44.7 ^a^)	**10h**	11 (NA ^a^)
9	**8i**	**7**	11.6 (NA ^a^)	**9i**	37.8 (52.1 ^a^)	**10i**	9 (NA ^a^)
10	**8j**	**7**	11 (22.2 ^a^)	**9j**	20.7 (40.9 ^a^)	**10j**	8 (NA ^a^)
11	**8k**	**7**	17.5 (14 ^a^)	**9k**	11.2 (48.6 ^a^)	**10k**	11 (NA ^a^)
12	**8l**	**7**	18.5 (25.4 ^a^)	**9l**	28.8 (39.5 ^a^)	**10l**	5 (NA ^a^)

^a^: Isolated yield by the improved method. NA = Was not obtained from column purification.

**Table 2:** DPPH and ABTS radical scavenging activity of compounds **9a**–**l**, curcumin and BHT.

Compounds	IC_50_ (μM) ^a^	
	DPPH Radical	ABTS Radical Cation
**9a**	37.57 ± 0.89 *	18.79 ± 1.58 *
**9b**	40.55 ± 6.04 *	22.05 ± 0.51 *
**9c**	49.22 ± 7.57 *	17.47 ± 1.09 *
**9d**	54.47 ± 4.08 *	46.17 ± 1.62
**9e**	37.17 ± 1.76 *	19.55 ± 0.08 *
**9f**	40.74 ± 0.97 **	17.91 ± 1.21 *
**9g**	40.47 ± 1.71 **	14.18 ± 1.44 *
**9h**	41.81 ± 3.11 *	11.36 ± 0.65 **
**9i**	48.12 ± 8.70 *	10.91 ± 0.77 **
**9j**	66.67 ± 8.60	22.80 ± 0.26 *
**9k**	42.18 ± 4.75 *	15.05 ± 0.01*
**9l**	63.77 ± 10.92 *	20.19 ± 1.08 *
curcumin	28.56 ± 4.12 **	10.14 ± 1.04 **
BHT ^b^	122.78 ± 0.96 *	71.94 ± 1.81 *

Results are presented as averages ± SD (n = 3). ^a^ Concentration necessary for 50% inhibition (IC_50_). ^b^ Butylated hydroxytoluene (BHT) was used as a positive control. * *p* < 0.05 compared with the control. ** *p* < 0.01 compared with the control.

## Data Availability

The data presented in this study are available in the main text and the Supplementary Materials of this article.

## Supplementary Materials

The following supporting information can be downloaded at: https://www.mdpi.com/article/10.3390/molecules27082547/s1. ^1^H and ^13^C NMR data for compounds **6**, **9a**–**l** and **10e**.
