# Supplementary material for: Improved Synthesis of Asymmetric Curcuminoids and Their Assessment as Antioxidants
Source: Molecules. 2022 Apr 14;27(8):2547. doi: 10.3390/molecules27082547 (PMC9030899; doi:10.3390/molecules27082547)
Supplement: Supplementary file 1 [file molecules-27-02547-s001.zip › molecules-1663635-supplementary.pdf]

Supporting materials

Improved Synthesis of Asymmetric Curcuminoids and their Assessment as Antioxidants

Yang-Je Cheng [1], Cai-Wei Li [2], Cing-Ling Kuo [1], Tzenge-Lien Shih [1,*], and Jih-Jung Chen [3,4,*]

[1] Department of Chemistry, Tamkang University, Tamsui Dist., New Taipei City 251301, Taiwan, Republic of China; jason0110422@gmail.com (Y.-J.C.); rose40635@gmail.com (C.-L.K.)

[2] Institute of Traditional Medicine, National Yang Ming Chiao Tung University, Taipei 112304, Taiwan; leecw1219.y@nycu.edu.tw (C.-W.L.)

[3] Department of Pharmacy, College of Pharmaceutical Sciences, National Yang Ming Chiao Tung University, Taipei 112304, Taiwan

[4] Department of Medical Research, China Medical University Hospital, China Medical University, Taichung 404332, Taiwan

* Correspondence: tlshih@mail.tku.edu.tw (T.-L.S.)*; jjungchen@nycu.edu.tw (J.-J.C.)*; Tel.: +886-2-2826-7195 (J.-J.C.)*; Fax: +886-2-2823-2940 (J.-J.C.)*.

Contents

Figure S1. ^{1}H NMR (600 MHz, $CDCl_3$) for compound **6**...S4

Figure S2. ^{13}C NMR (150 MHz, $CDCl_3$) for compound **6**...S4

Figure S3. ^{1}H NMR (600 MHz, $CDCl_3$) for compound **9a**...S5

Figure S4. ^{13}C NMR (150 MHz, $CDCl_3$) for compound **9a**...S5

Figure S5. ^{1}H NMR (600 MHz, $CDCl_3$) for compound **9b**...S6

Figure S6. ^{13}C NMR (150 MHz, $CDCl_3$) for compound **9b**...S6

Figure S7. ^{1}H NMR (600 MHz, $CDCl_3$) for compound **9c**...S7

Figure S8. ^{13}C NMR (150 MHz, $CDCl_3$) for compound **9c**...S7

Figure S9. ^{1}H NMR (600 MHz, $CDCl_3$) for compound **9d**...S8

Figure S10. ^{13}C NMR (150 MHz, $CDCl_3$) for compound **9d**...S8

Figure S11. ^{1}H NMR (600 MHz, CD_3OD) for compound **9e**...S9

Figure S12. ^{13}C NMR (150 MHz, CD_3OD) for compound **9e**...S9

Figure S13. ^{1}H NMR (600 MHz, CD_3OD) for compound **9f**...S10

Figure S14. ^{13}C NMR (150 MHz, CD_3OD) for compound **9f**...S10

Figure S15. ^{1}H NMR (600 MHz, $CDCl_3$) for compound **9g**...S11

Figure S16. ^{13}C NMR (150 MHz, $CDCl_3$) for compound **9g**...S11

Figure S17. ^{1}H NMR (600 MHz, $CDCl_3$) for compound **9h**...S12

Figure S18. ^{13}C NMR (150 MHz, $CDCl_3$) for compound **9h**...S12

Figure S19. ^{1}H NMR (600 MHz, $CDCl_3$) for compound **9i**...S13

Figure S20. ^{13}C NMR (150 MHz, $CDCl_3$) for compound **9i**...S13

Figure S21. ^{1}H NMR (600 MHz, CD_3OD) for compound **9j**...S14

Figure S22. ^{13}C NMR (150 MHz, CD_3OD) for compound **9j**...S14

Figure S23. ^{1}H NMR (600 MHz, $CDCl_3$) for compound **9k**....S15

Figure S24. ^{13}C NMR (150 MHz, $CDCl_3$) for compound **9k**....S15

Figure S25. ^{1}H NMR (600 MHz, $CDCl_3$) for compound **9l**....S16

Figure S26. ^{13}C NMR (150 MHz, $CDCl_3$) for compound **9l**....S16

Figure S27. ^{1}H NMR (600 MHz, $CDCl_3$) for compound **10e**....S17

Figure S28. ^{13}C NMR (150 MHz, $CDCl_3$) for compound **10e**....S17

Figure S29. HR-ESI-MS for compound **9a**....S18

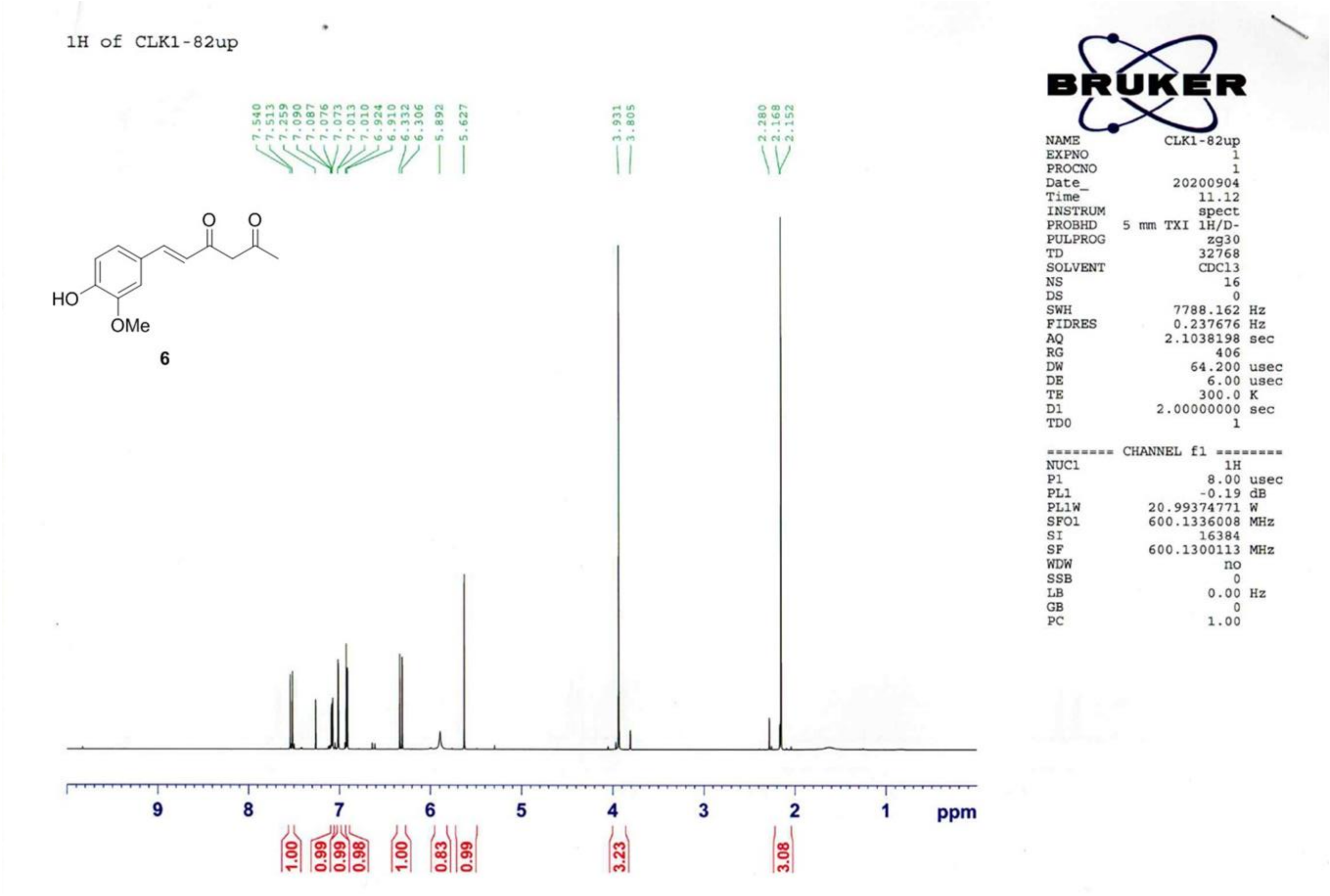

Figure S1. ^{1}H NMR (600 MHz, $CDCl_3$) for compound **6.**

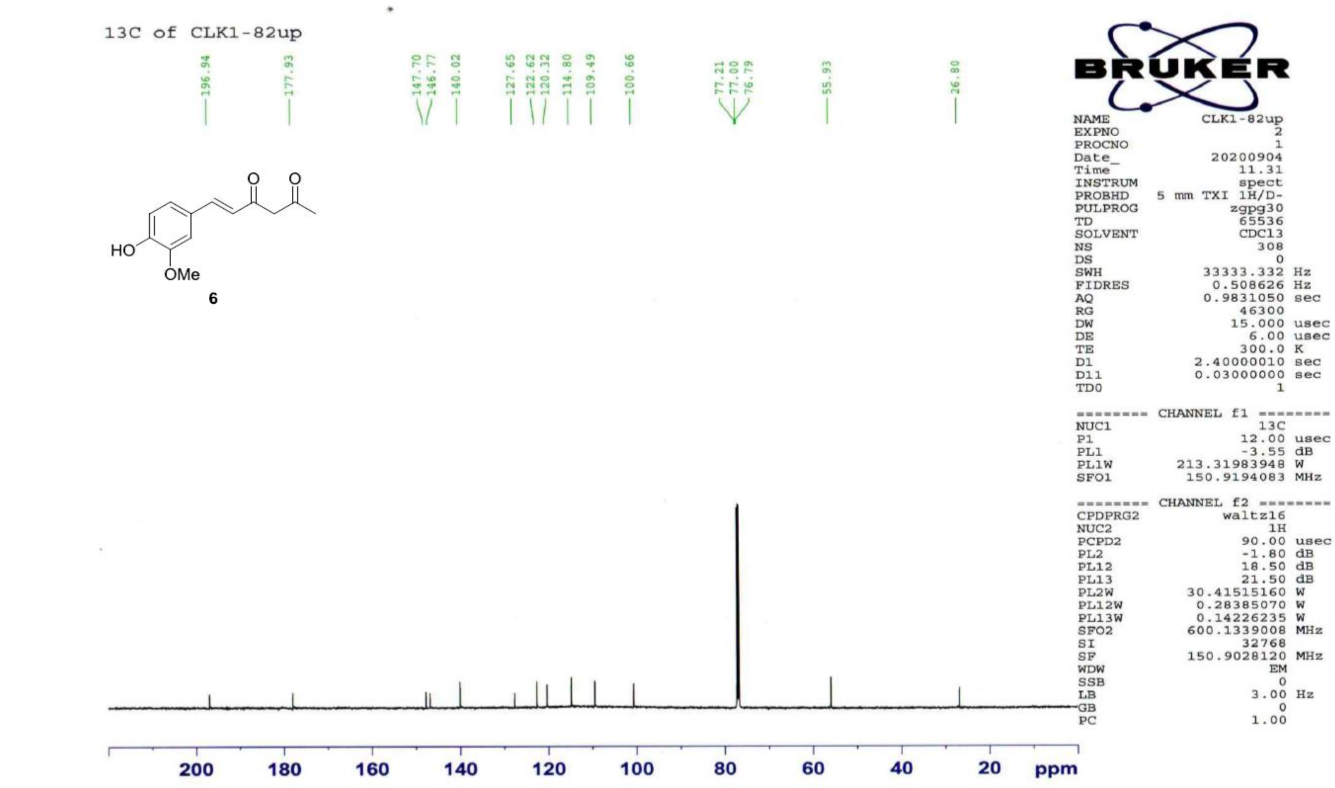

Figure S2. ^{13}C NMR (150 MHz, $CDCl_3$) for compound **6.**

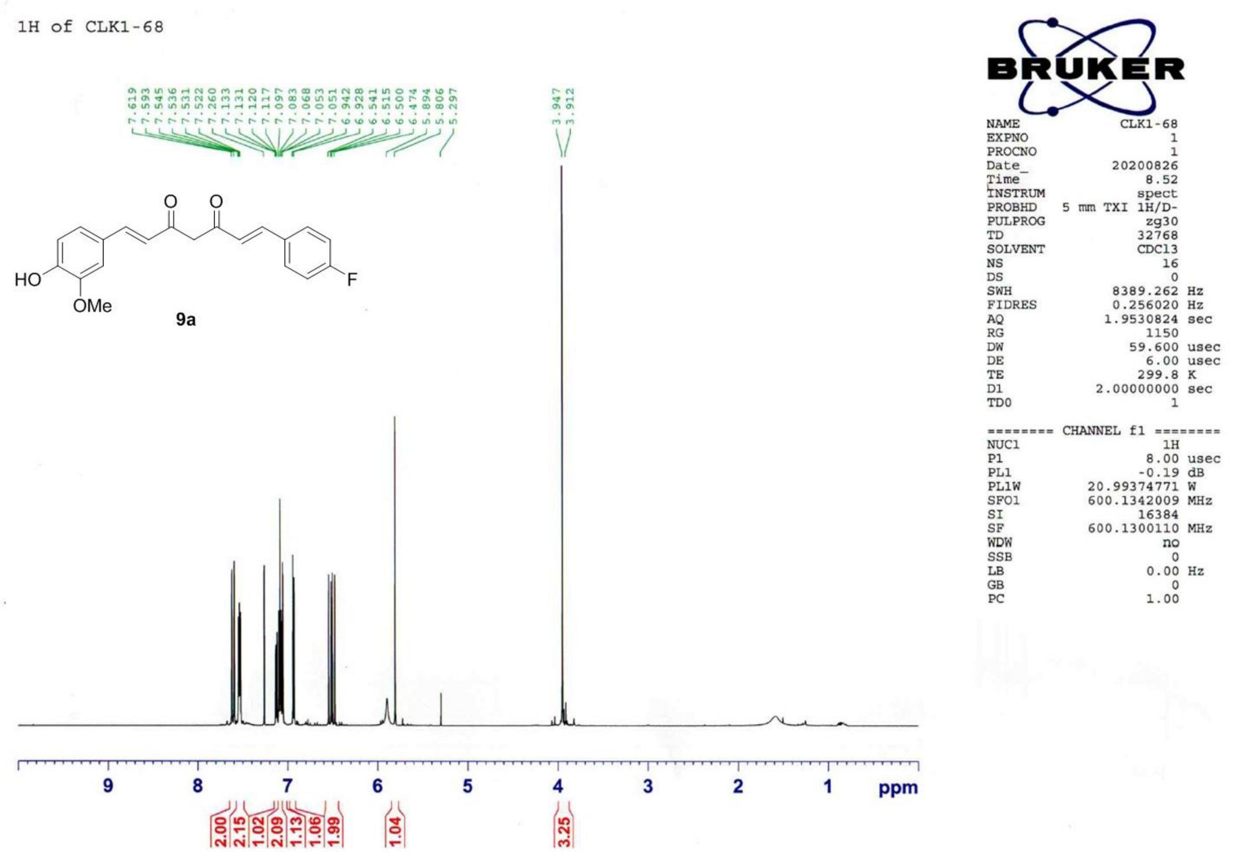

Figure S3. ^{1}H NMR (600 MHz, $CDCl_3$) for compound **9a.**

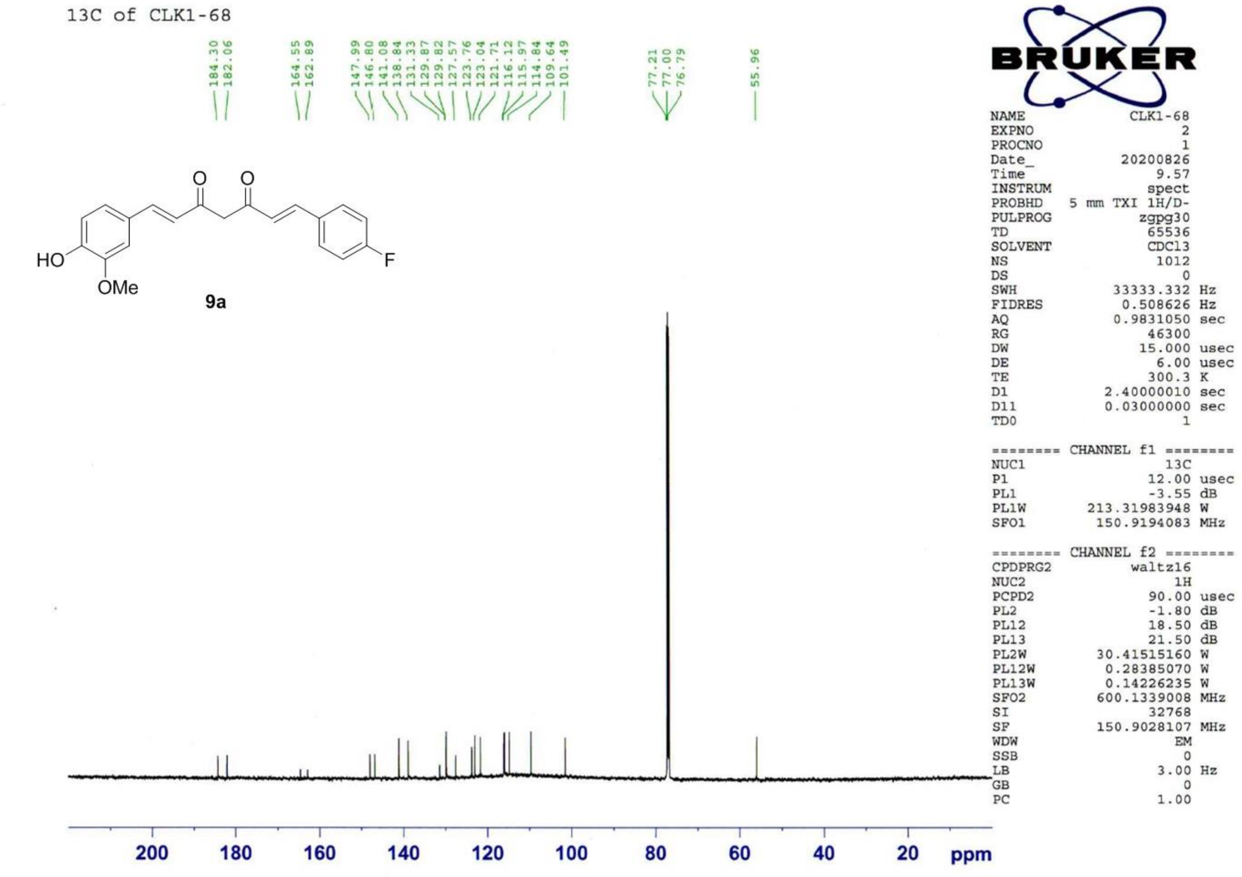

Figure S4. ^{13}C NMR (150 MHz, $CDCl_3$) for compound **9a.**

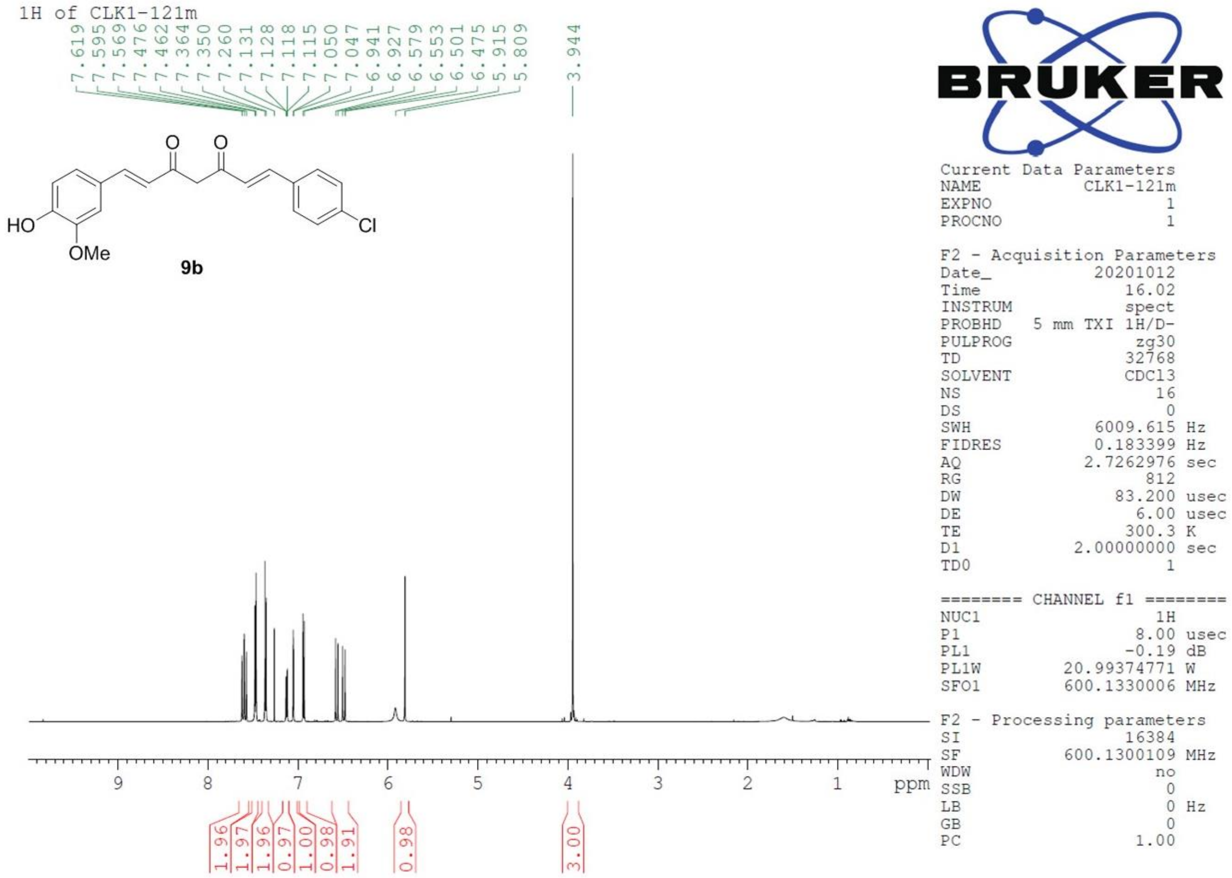

Figure S5. ^{1}H NMR (600 MHz, $CDCl_3$) for compound **9b.**

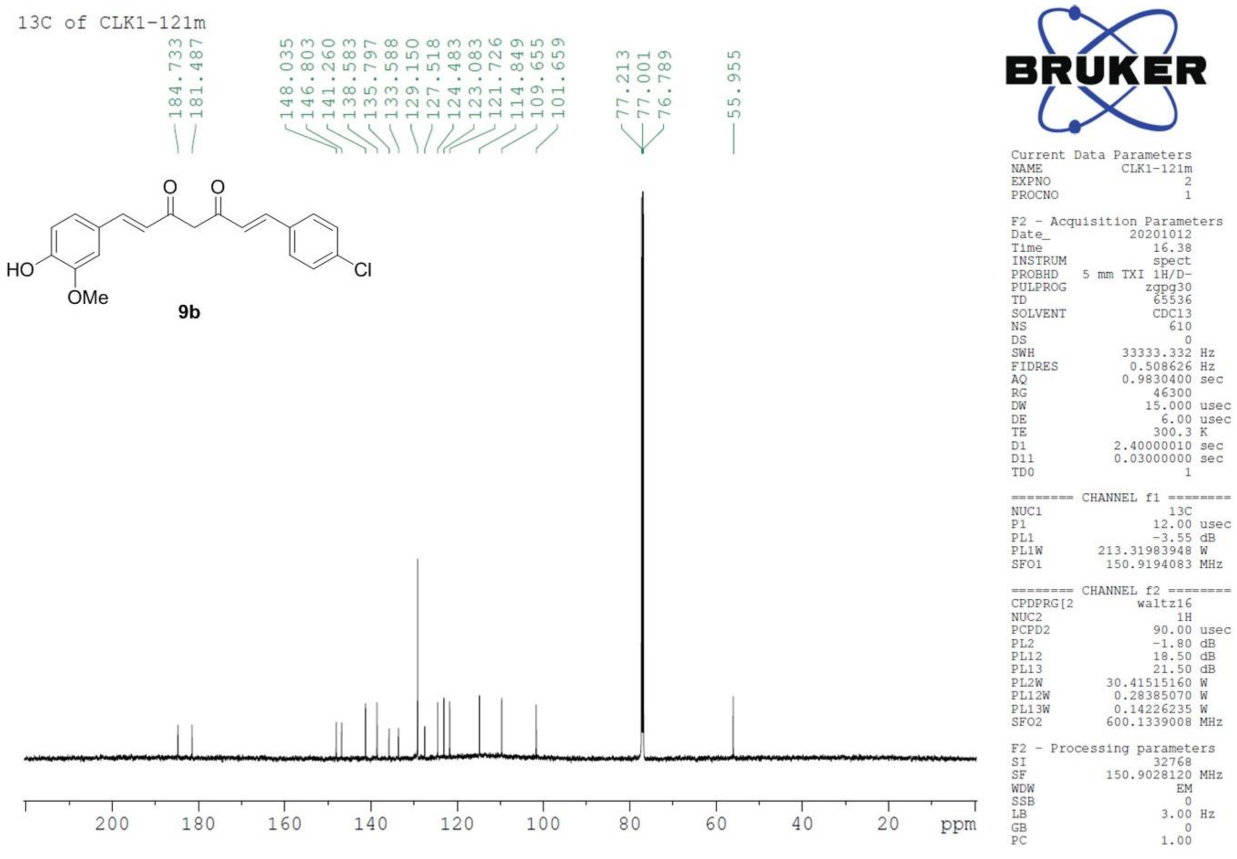

Figure S6. ^{13}C NMR (150 MHz, $CDCl_3$) for compound **9b**.

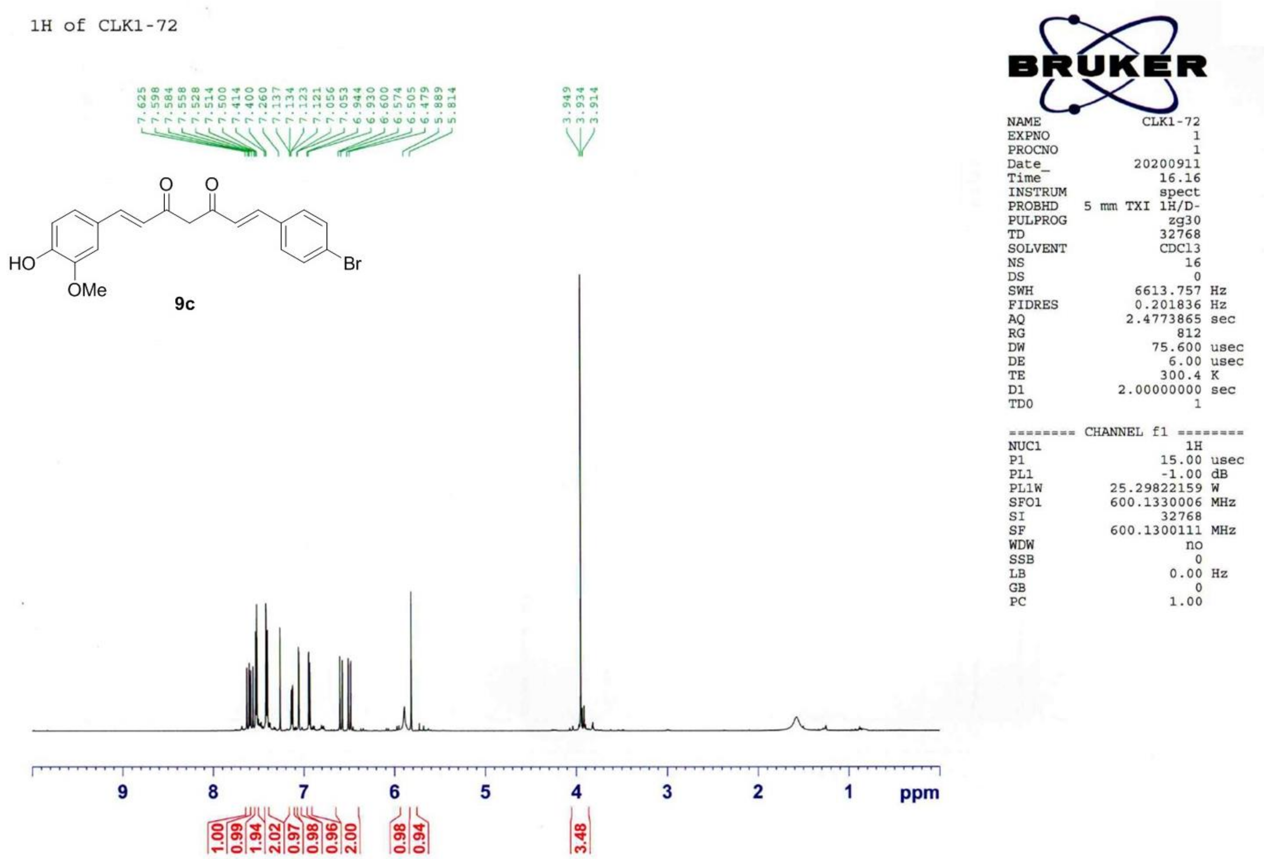

Figure S7. ^{1}H NMR (600 MHz, $CDCl_3$) for compound **9c.**

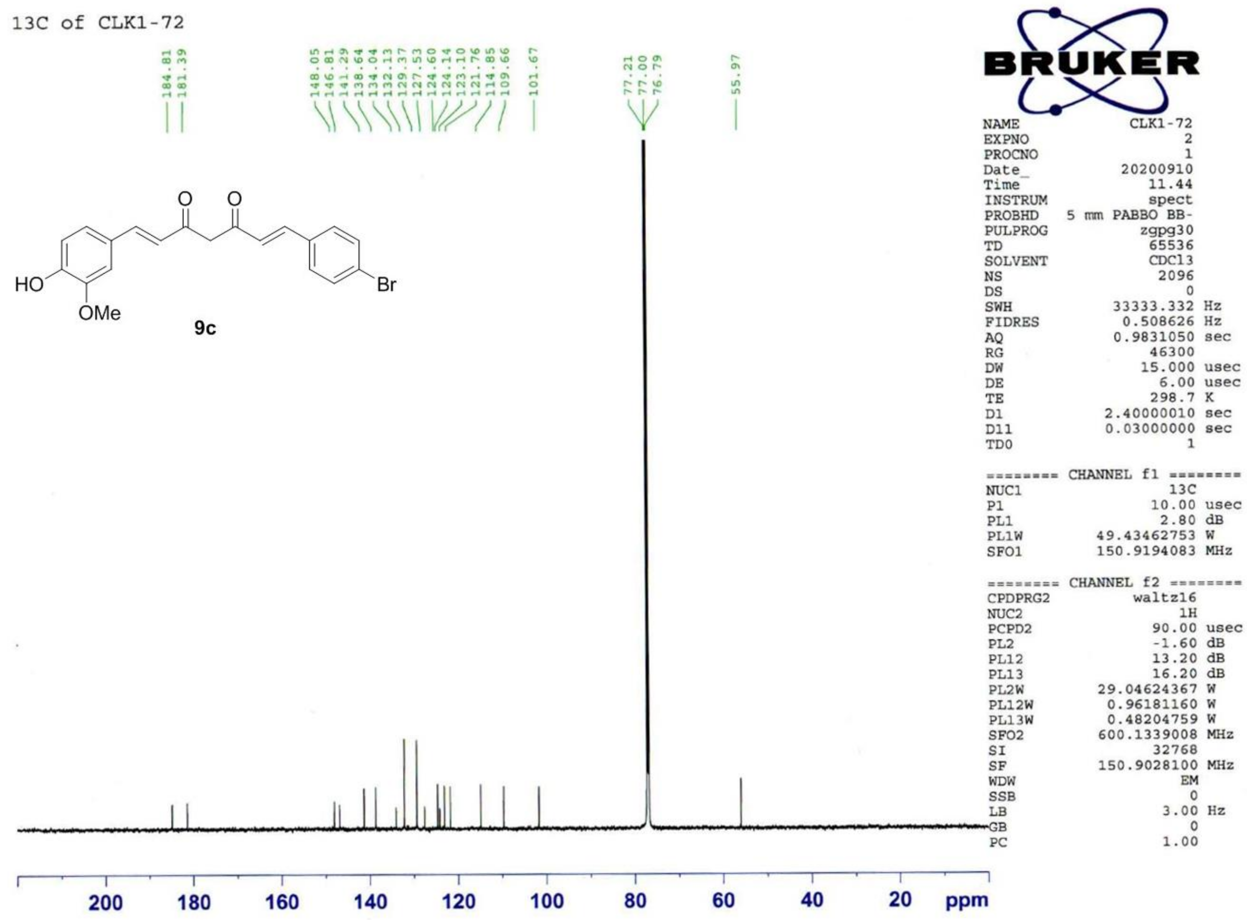

Figure S8. ^{13}C NMR (150 MHz, $CDCl_3$) for compound **9c.**

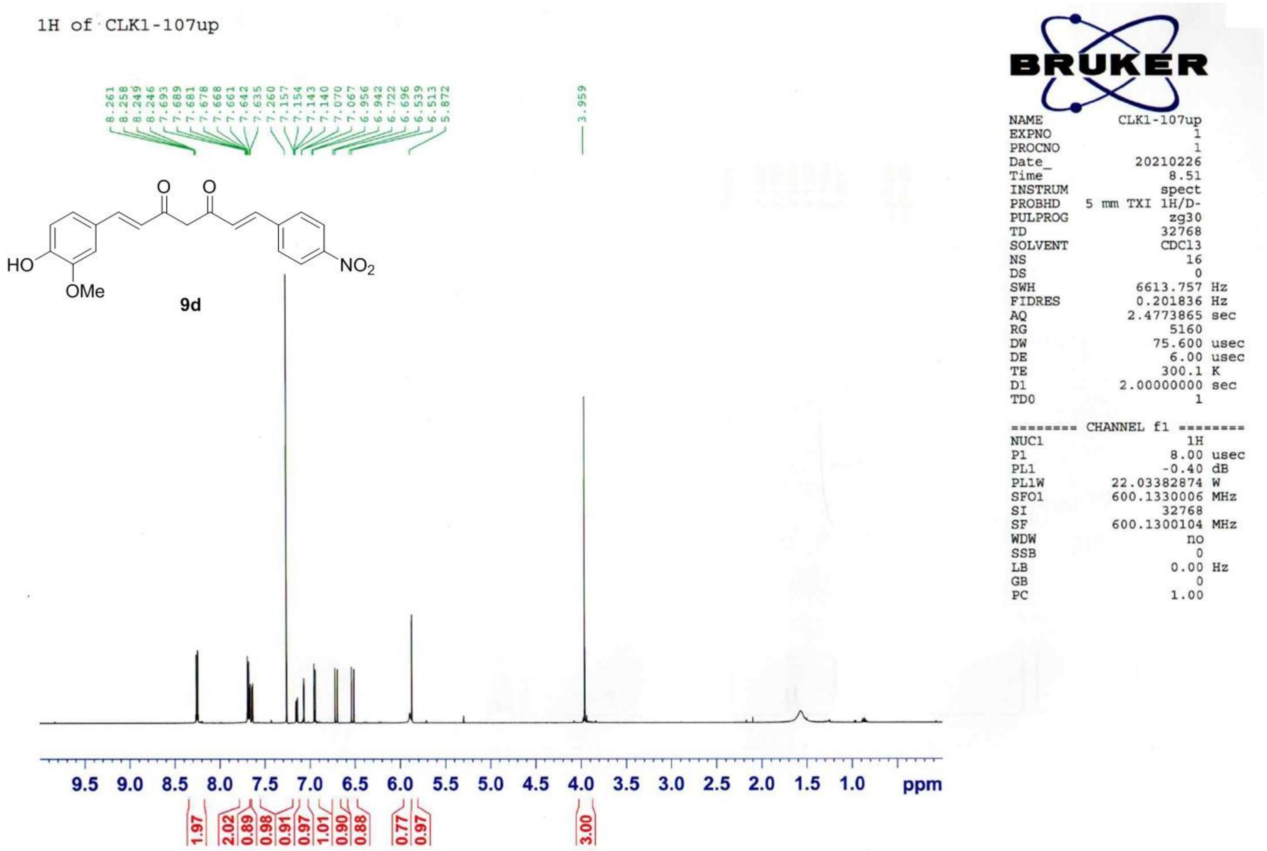

Figure S9. ^{1}H NMR (600 MHz, $CDCl_3$) for compound **9d.**

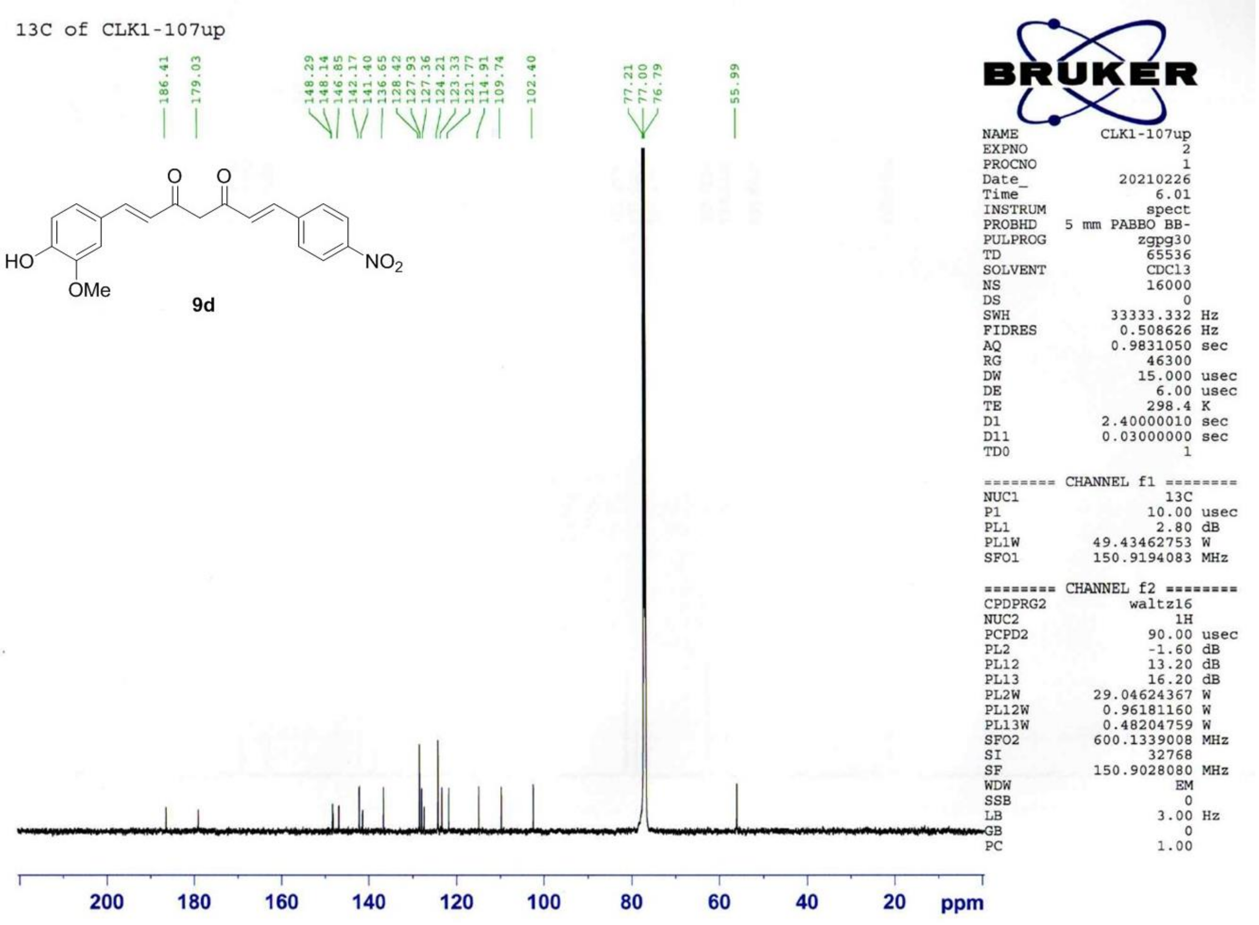

Figure S10. ^{13}C NMR (150 MHz, $CDCl_3$) for compound **9d.**

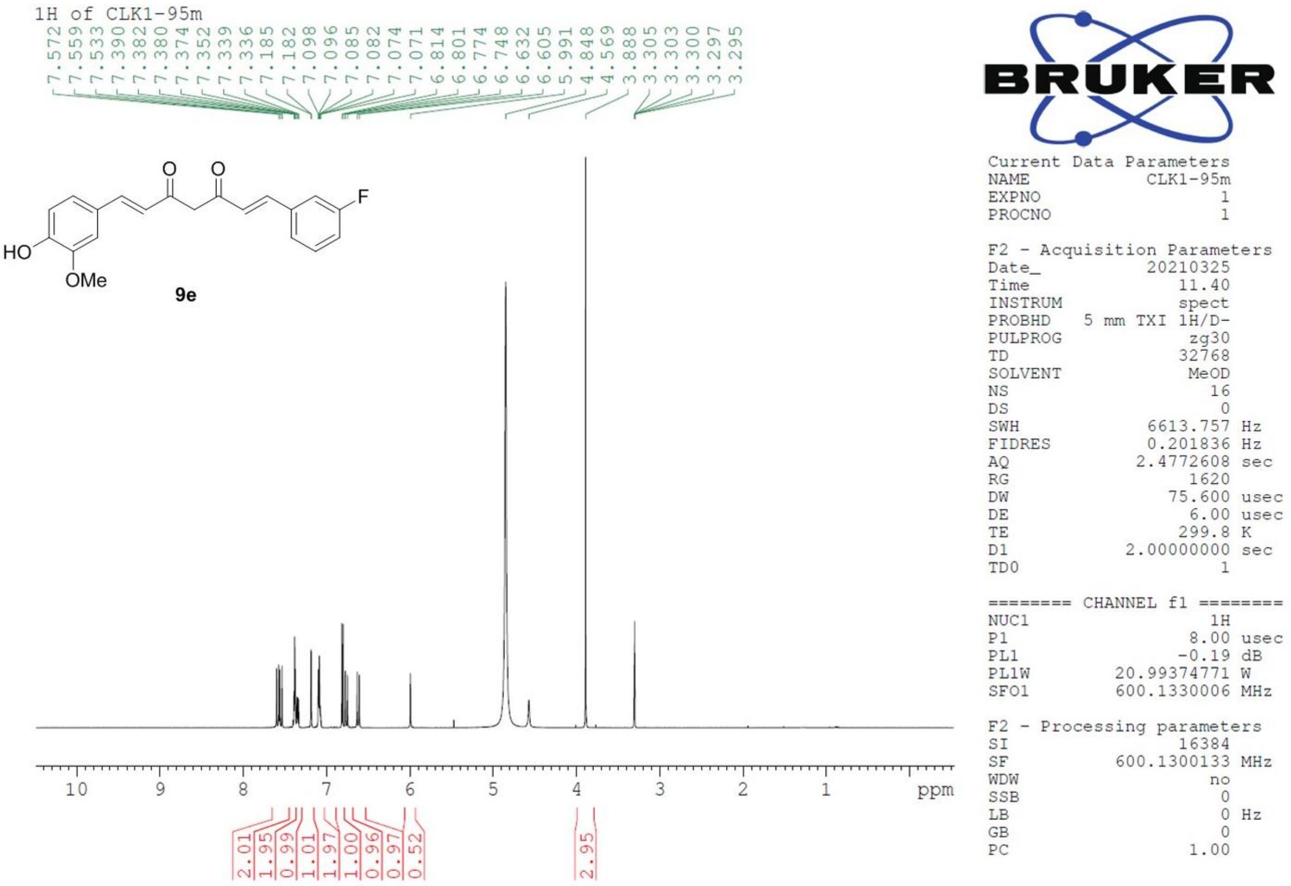

Figure S11. ^{1}H NMR (600 MHz, CD_3OD) for compound **9e.**

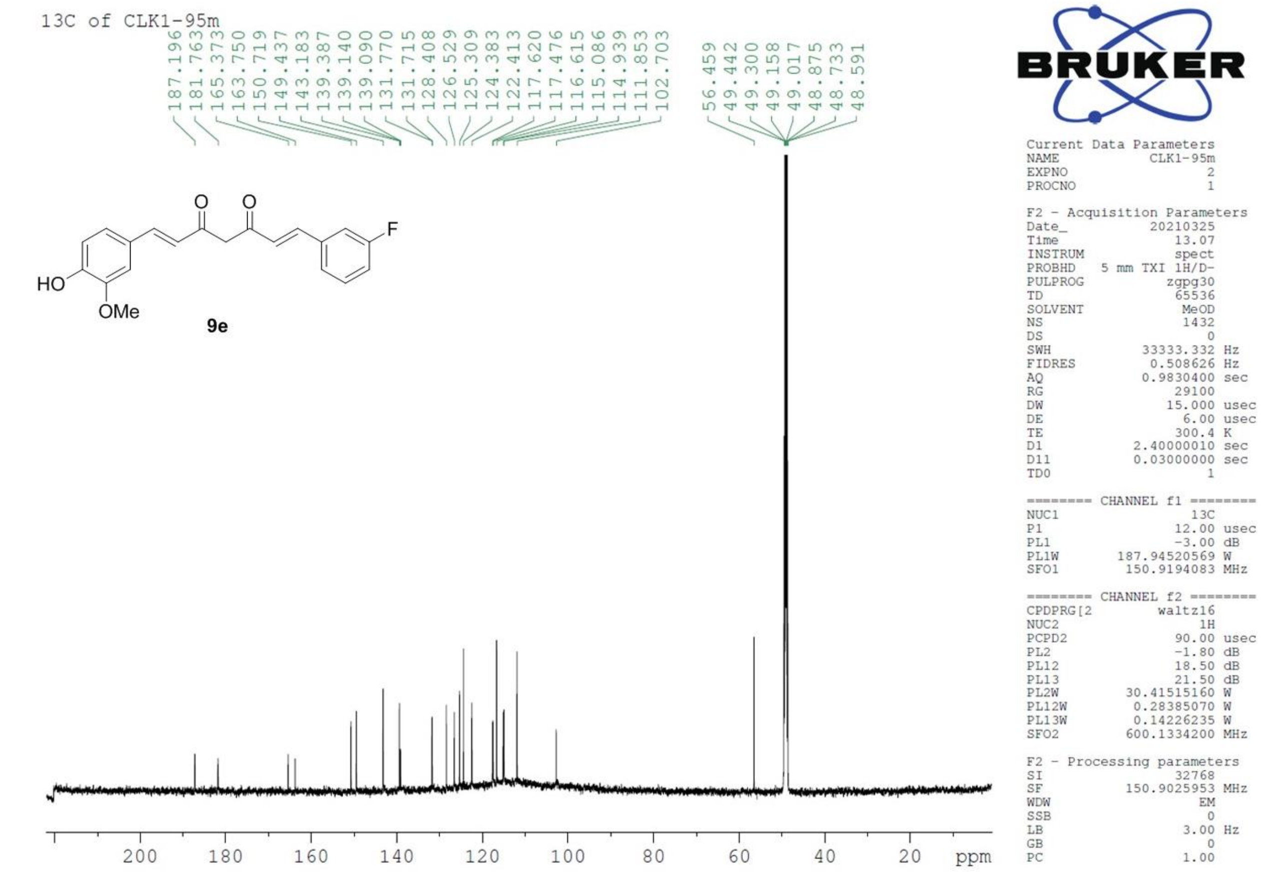

Figure S12. ^{13}C NMR (150 MHz, CD_3OD) for compound **9e**.

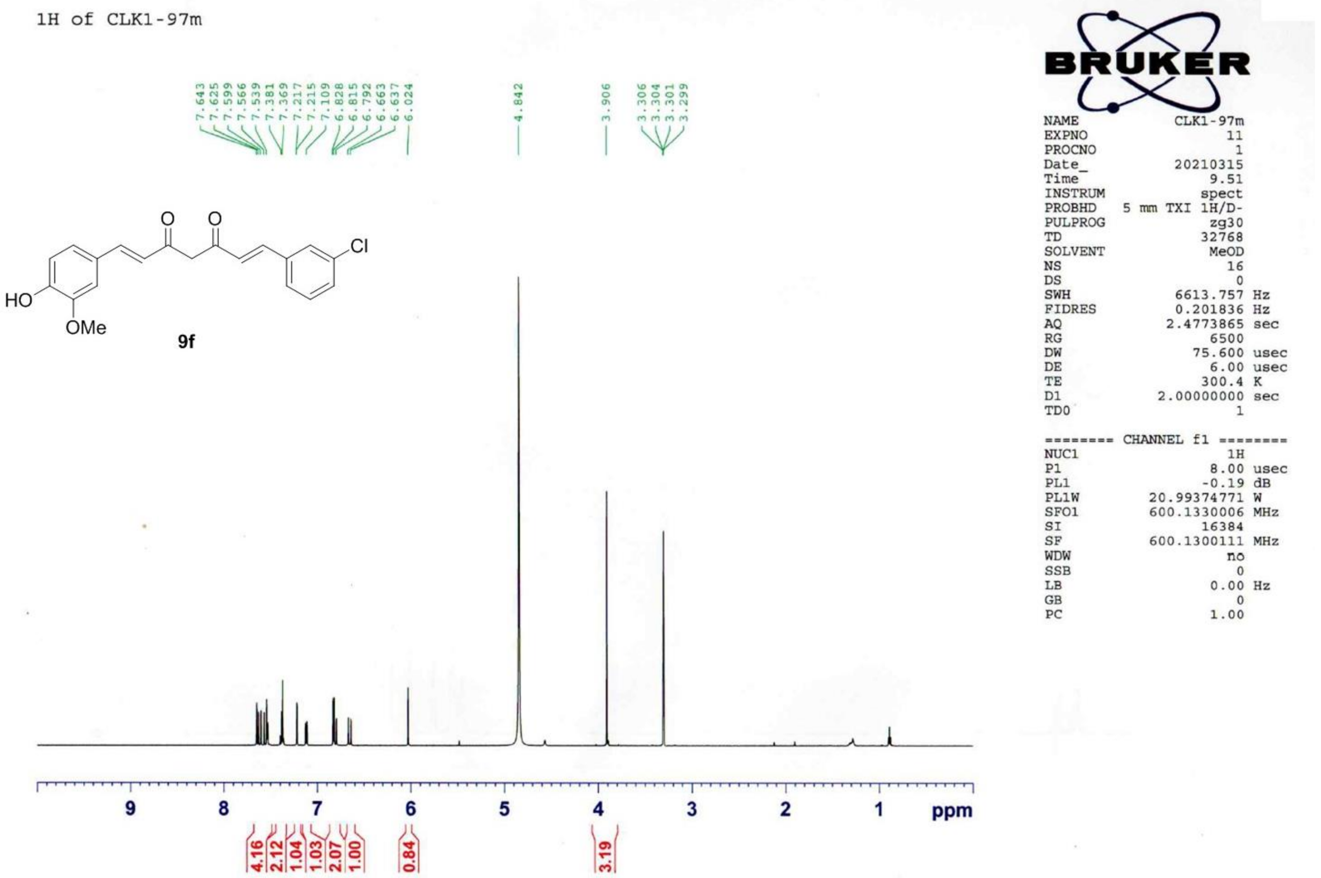

Figure S13. ^{1}H NMR (600 MHz, CD_3OD) for compound **9f**.

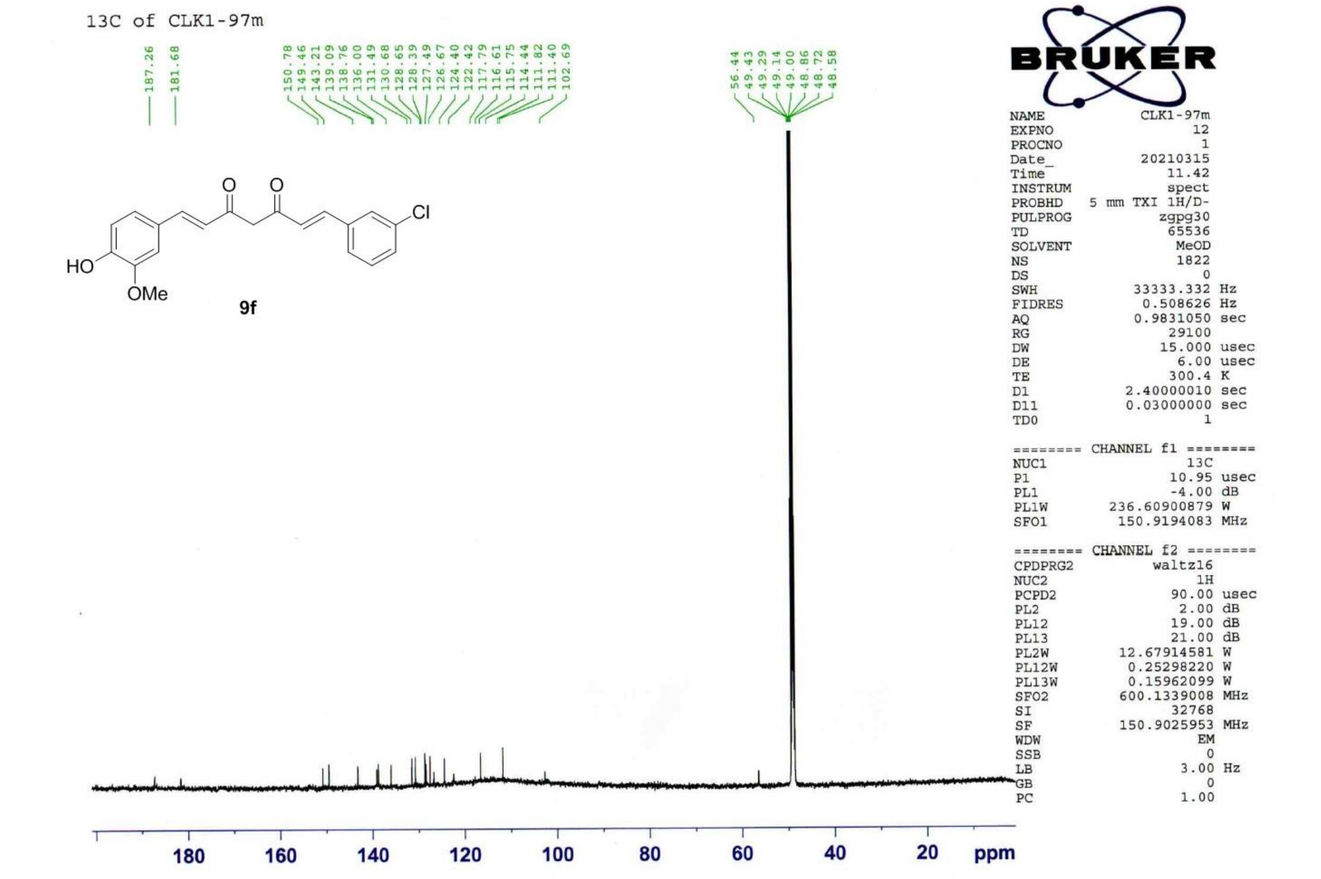

Figure S14. ^{13}C NMR (150 MHz, CD_3OD) for compound **9f.**

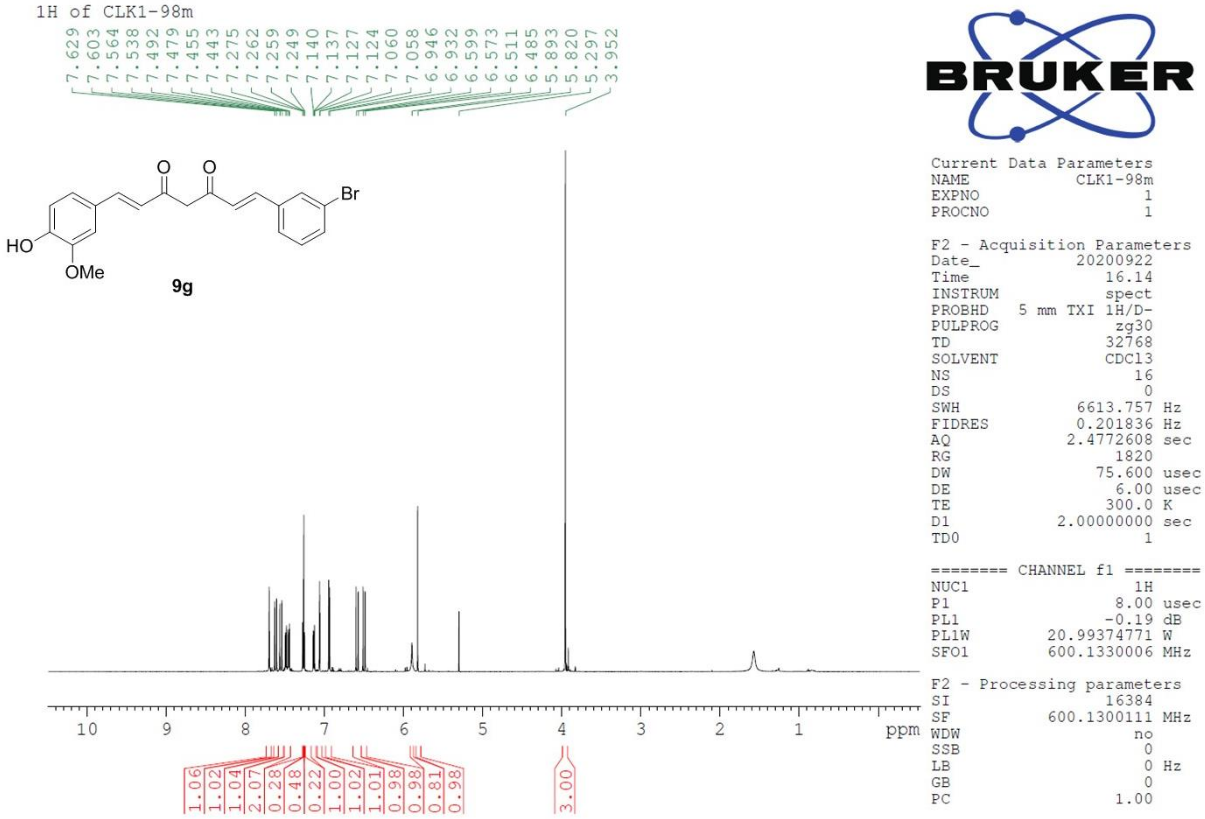

Figure S15. ^{1}H NMR (600 MHz, $CDCl_3$) for compound **9g.**

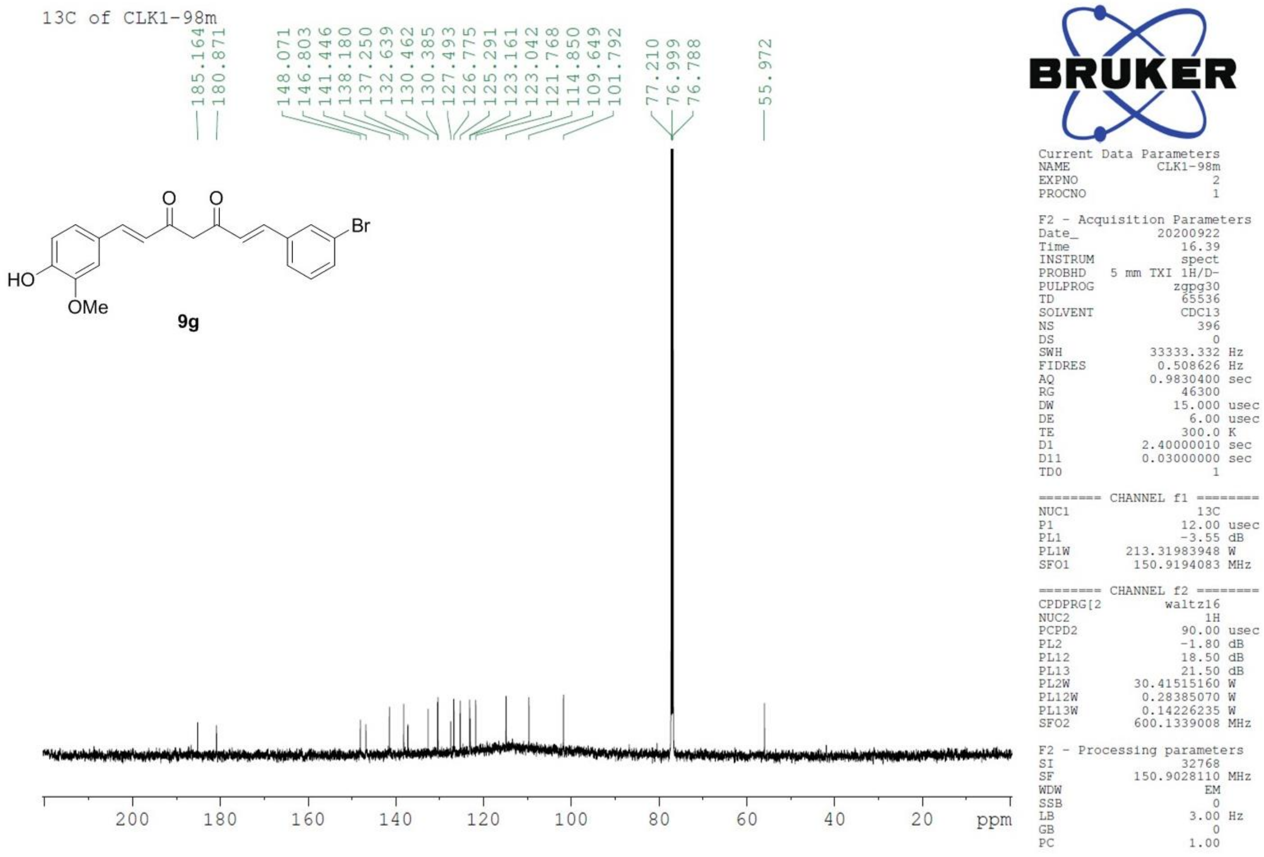

Figure S16. ^{13}C NMR (150 MHz, $CDCl_3$) for compound **9g**.

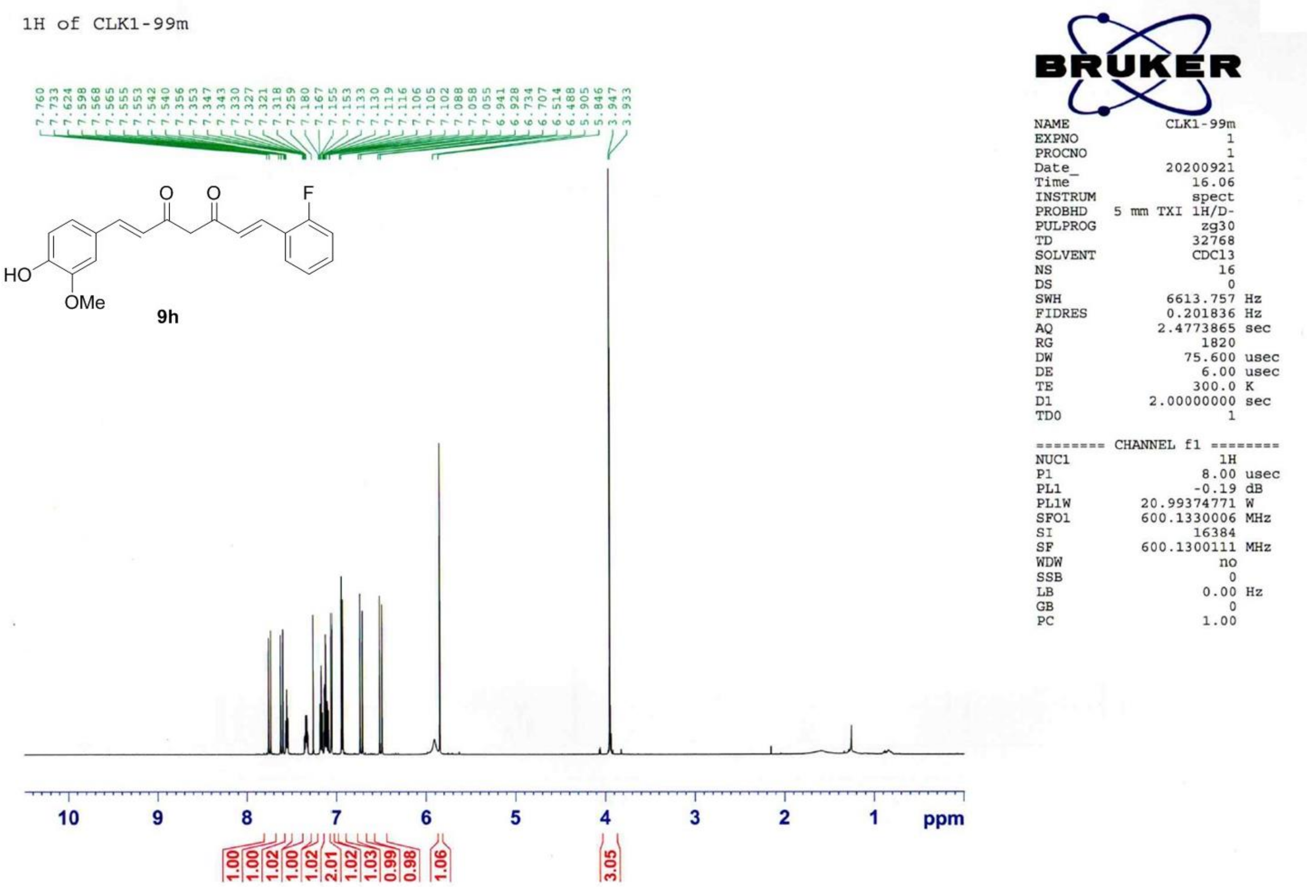

Figure S17. ^{1}H NMR (600 MHz, $CDCl_3$) for compound **9h**.

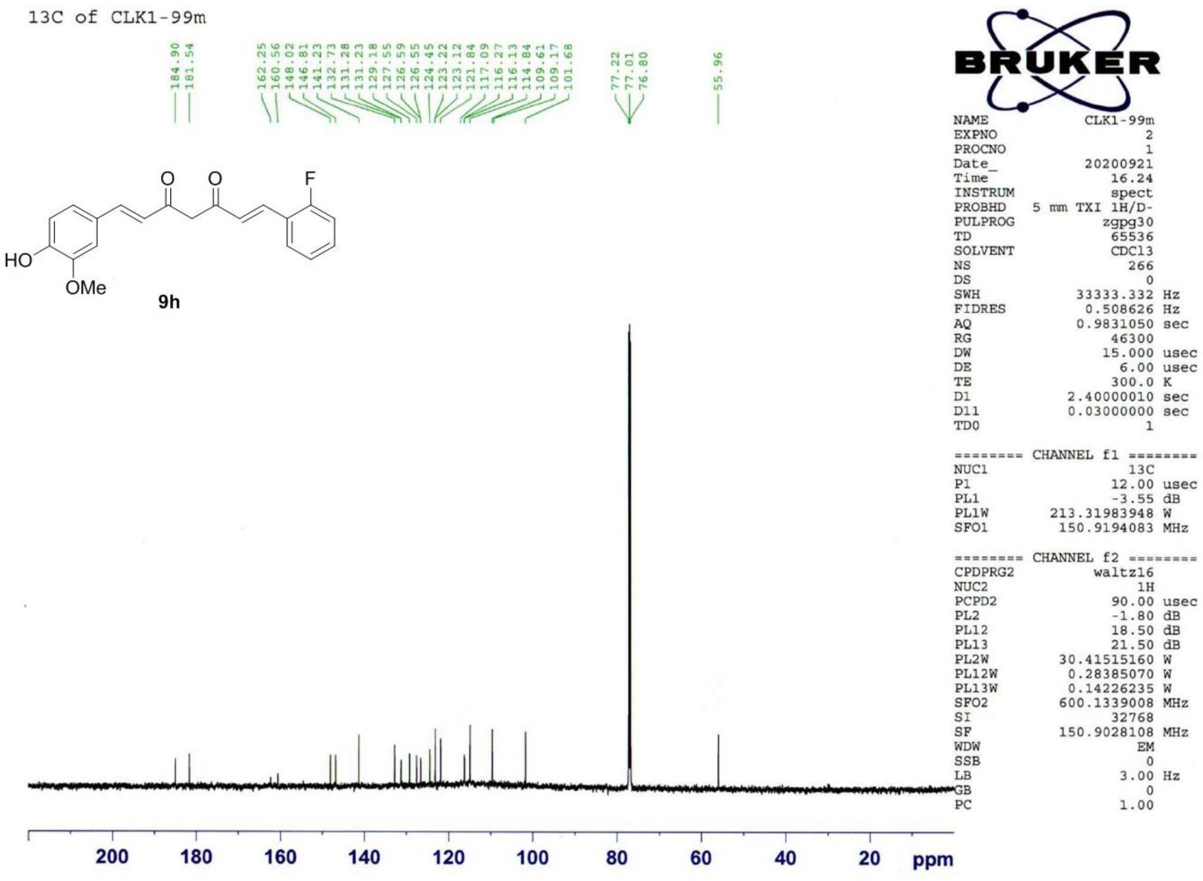

Figure S18. ^{13}C NMR (150 MHz, $CDCl_3$) for compound **9h**.

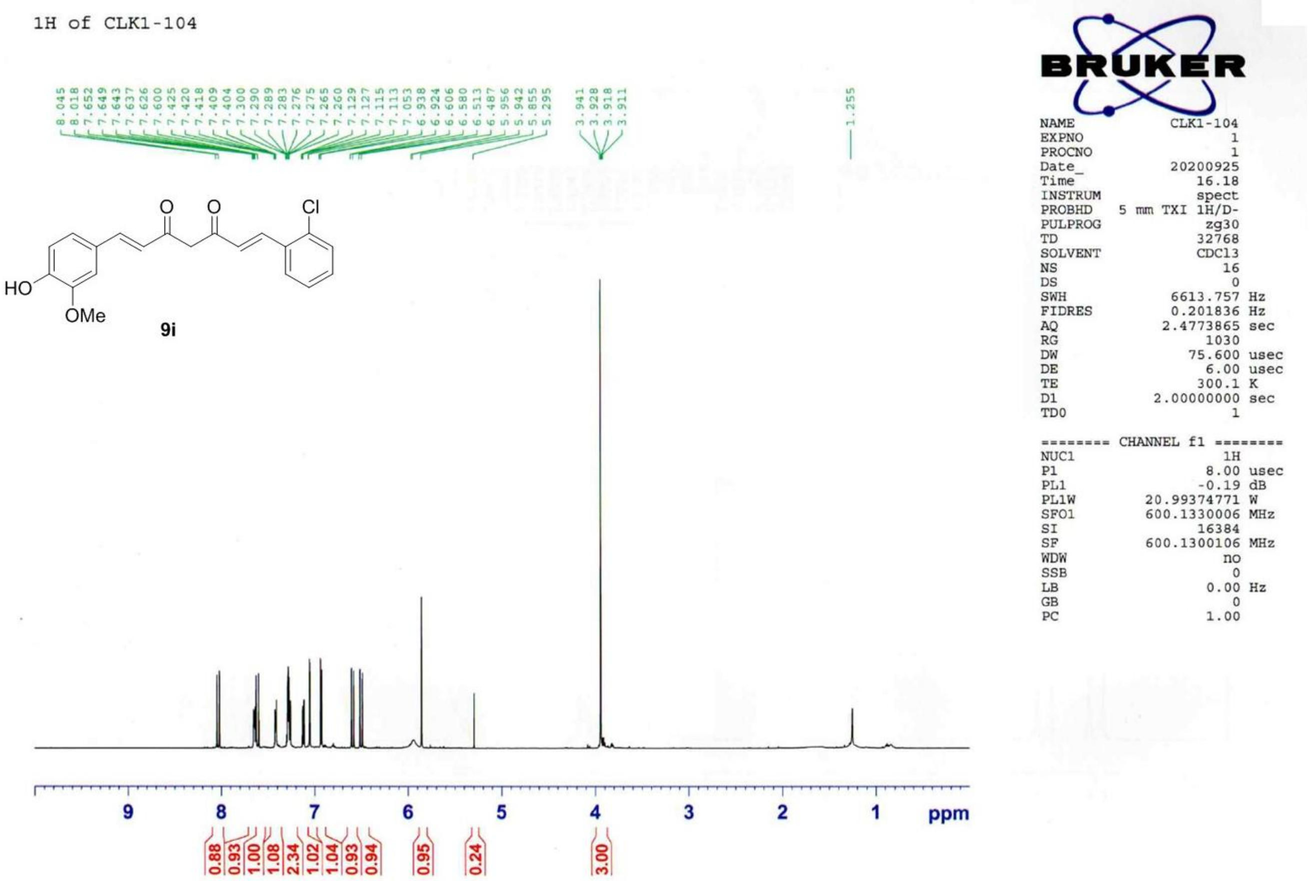

Figure S19. ^{1}H NMR (600 MHz, $CDCl_3$) for compound **9i**.

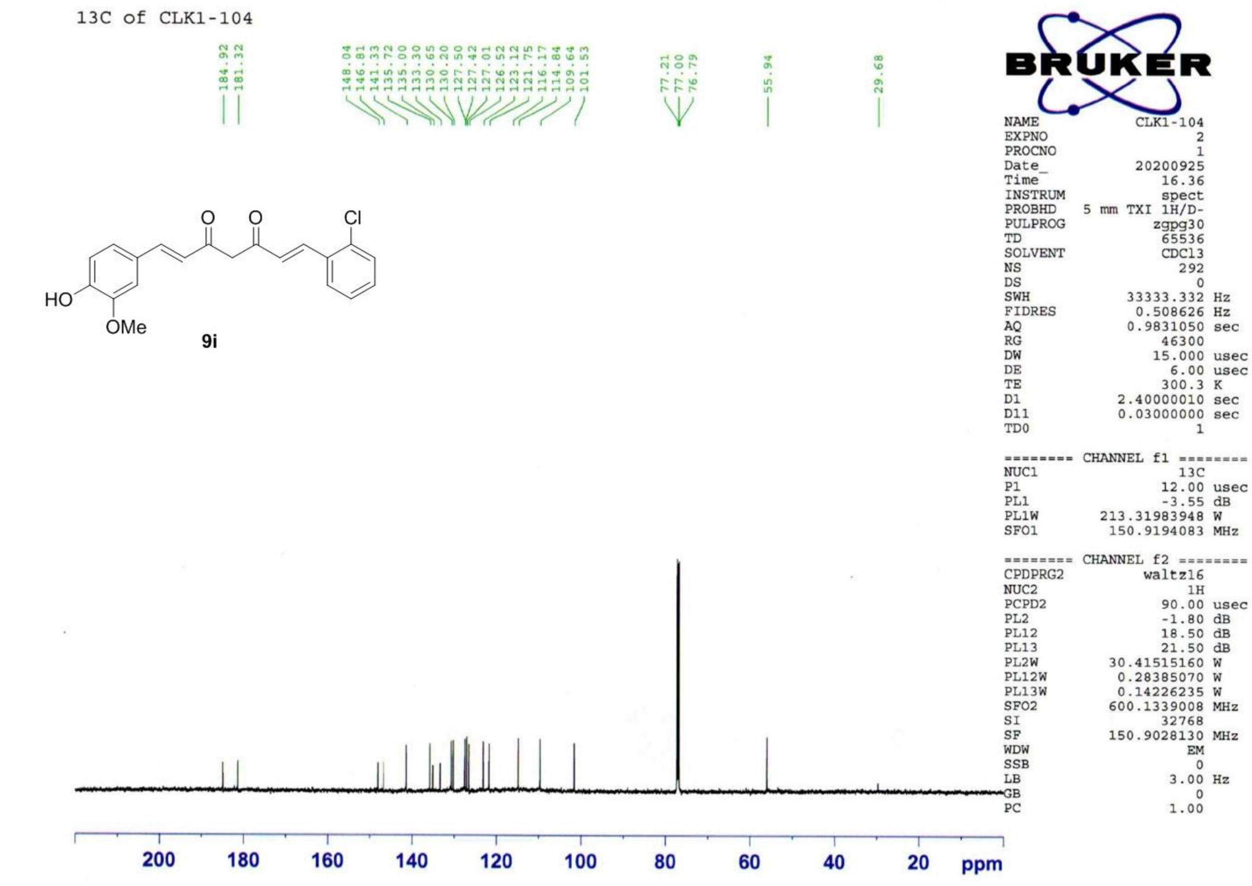

Figure S20. ^{13}C NMR (150 MHz, $CDCl_3$) for compound **9i**.

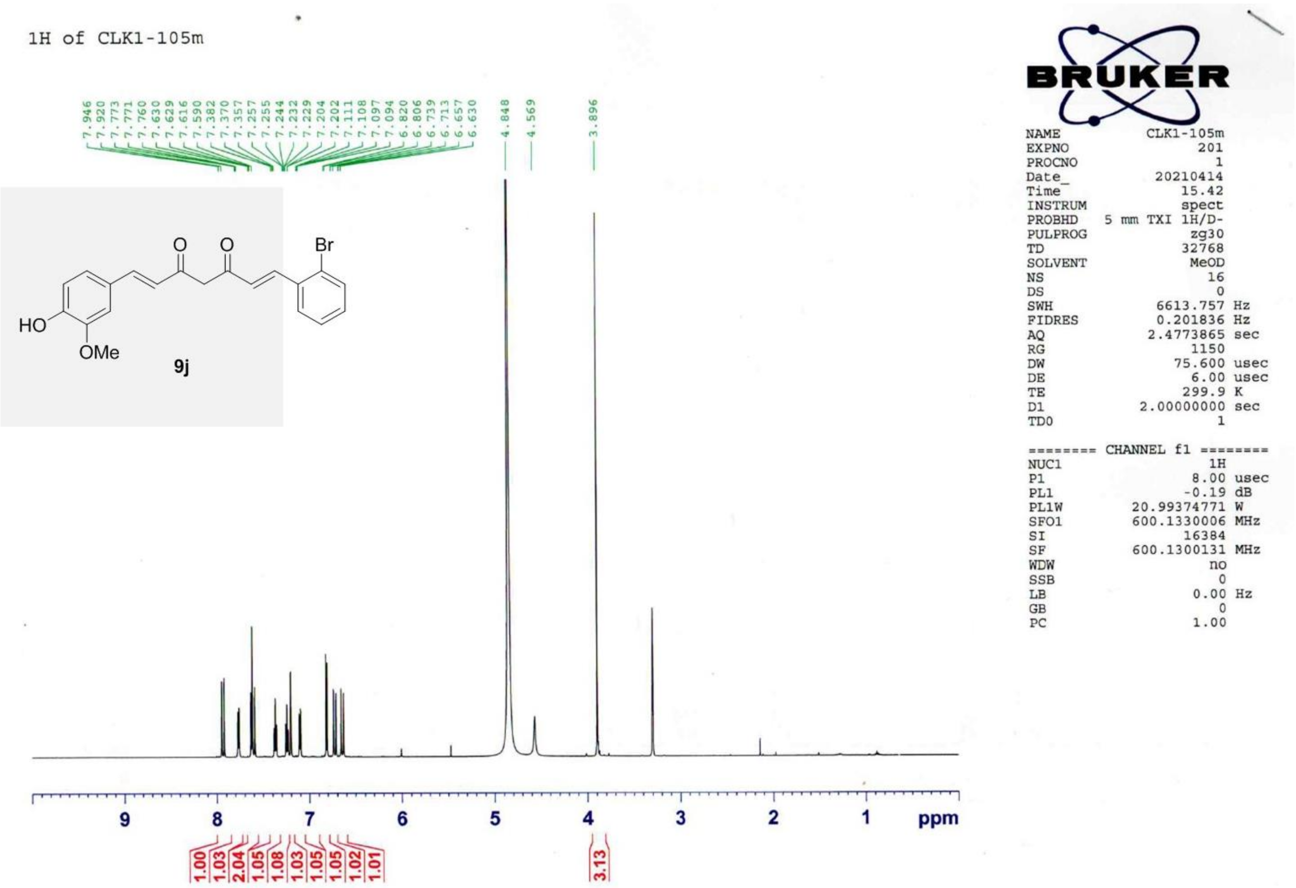

Figure S21. ^{1}H NMR (600 MHz, CD_3OD) for compound **9j**.

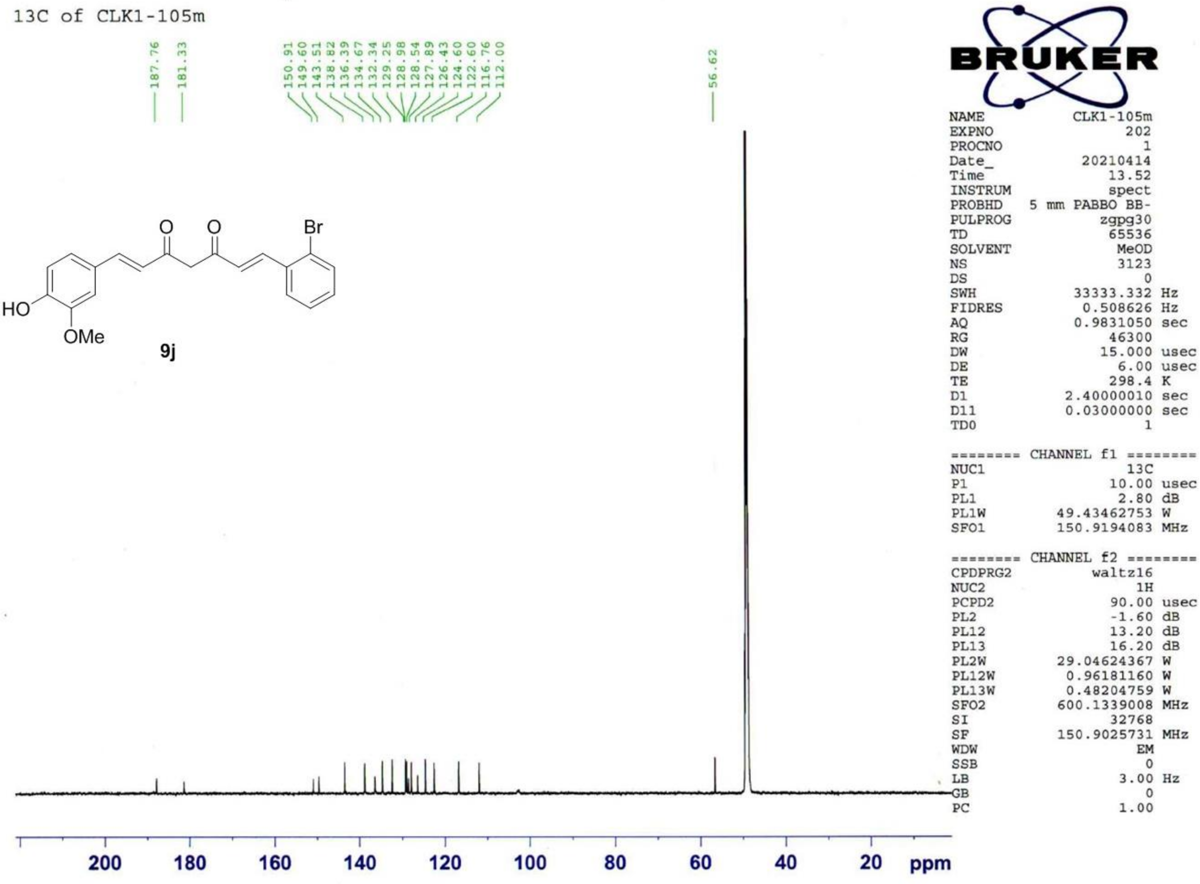

Figure S22. ^{13}C NMR (150 MHz, CD_3OD) for compound **9j**.

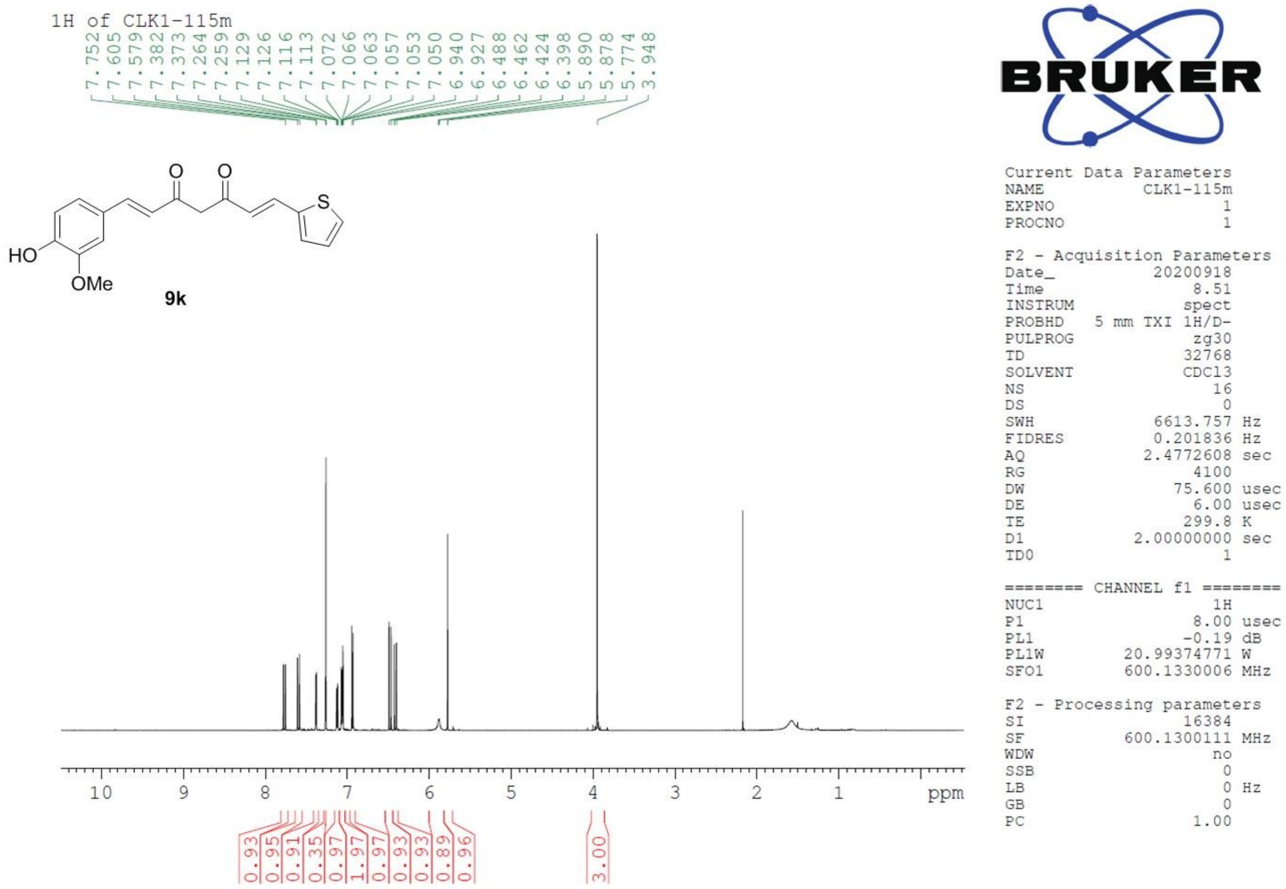

Figure S23. ^{1}H NMR (600 MHz, $CDCl_3$) for compound **9k**.

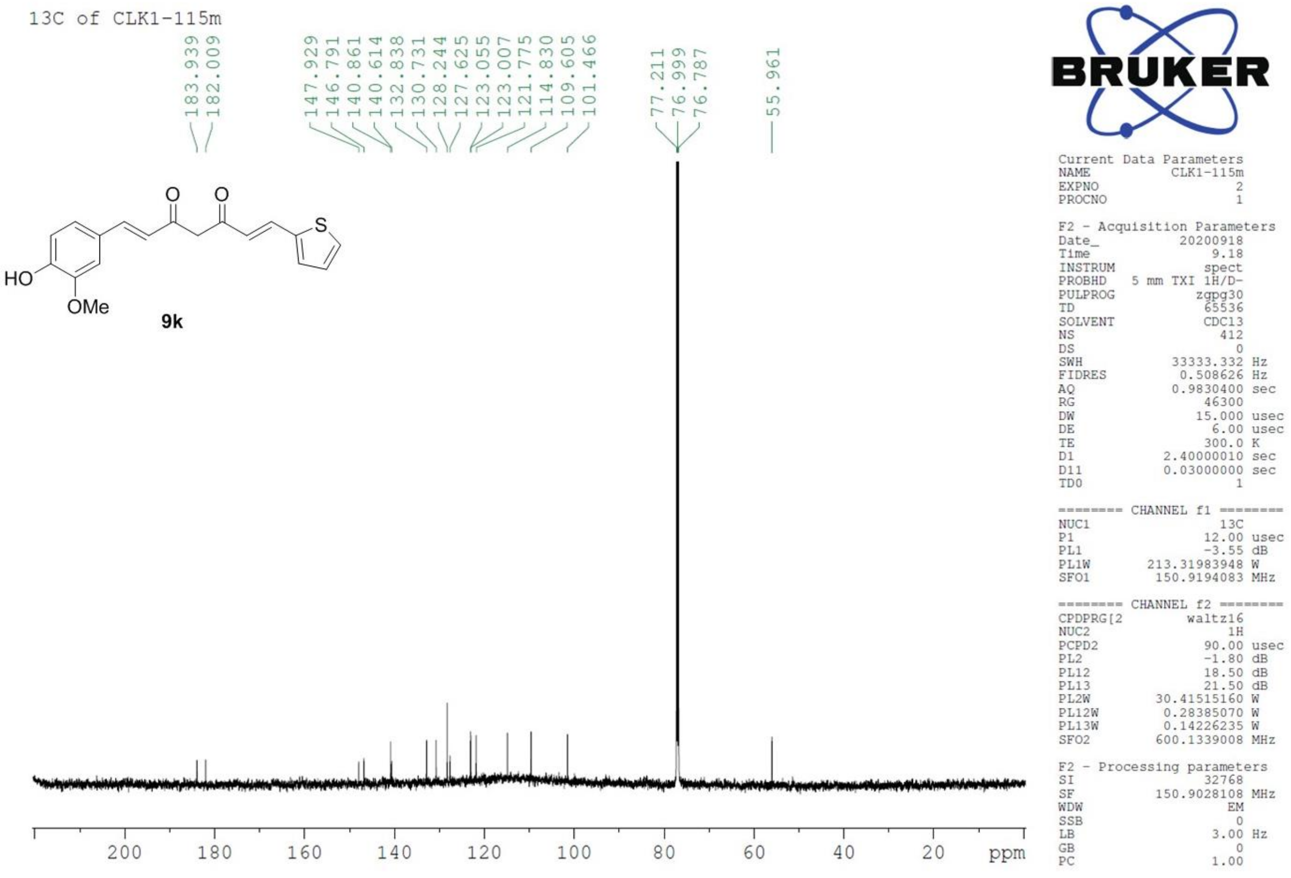

Figure S24. ^{13}C NMR (150 MHz, $CDCl_3$) for compound **9k.**

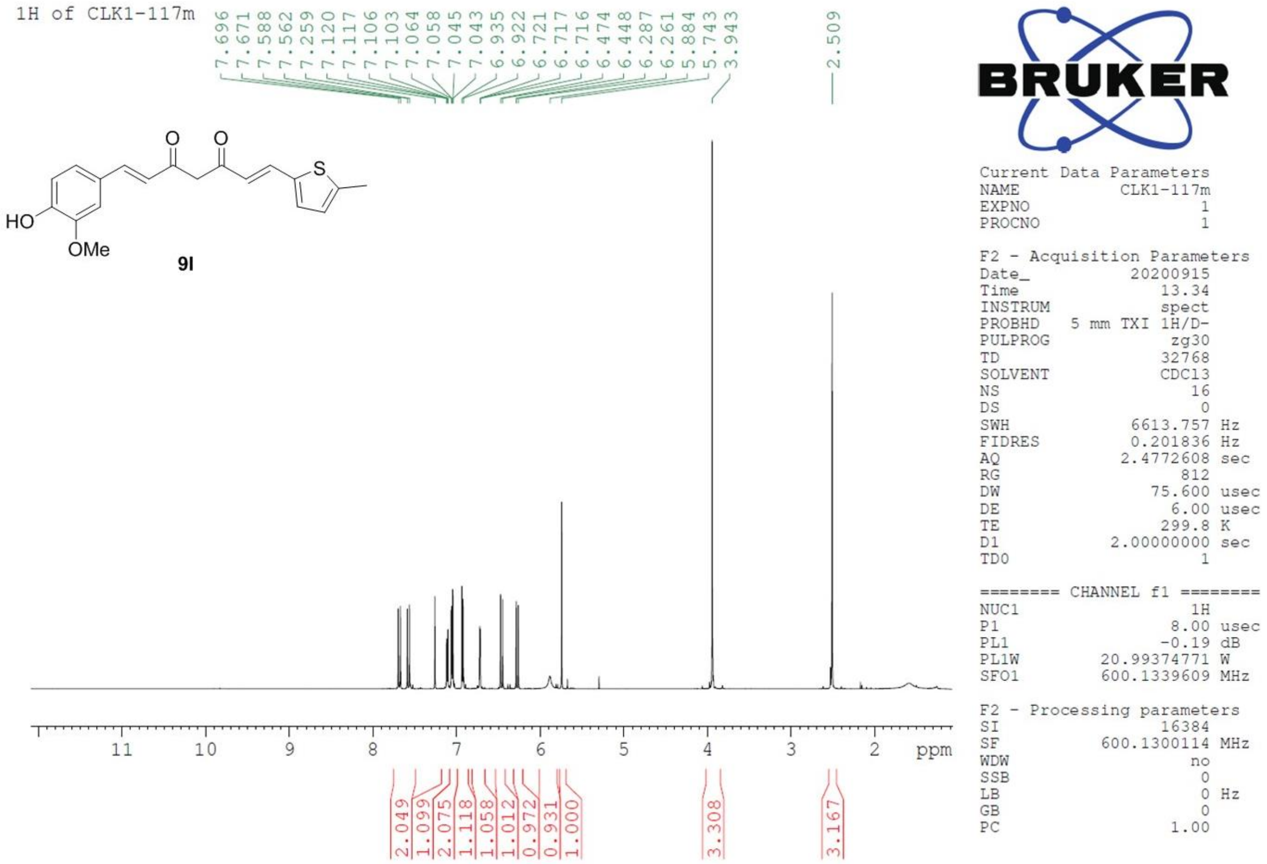

Figure S25. ^{1}H NMR (600 MHz, $CDCl_3$) for compound **9l**.

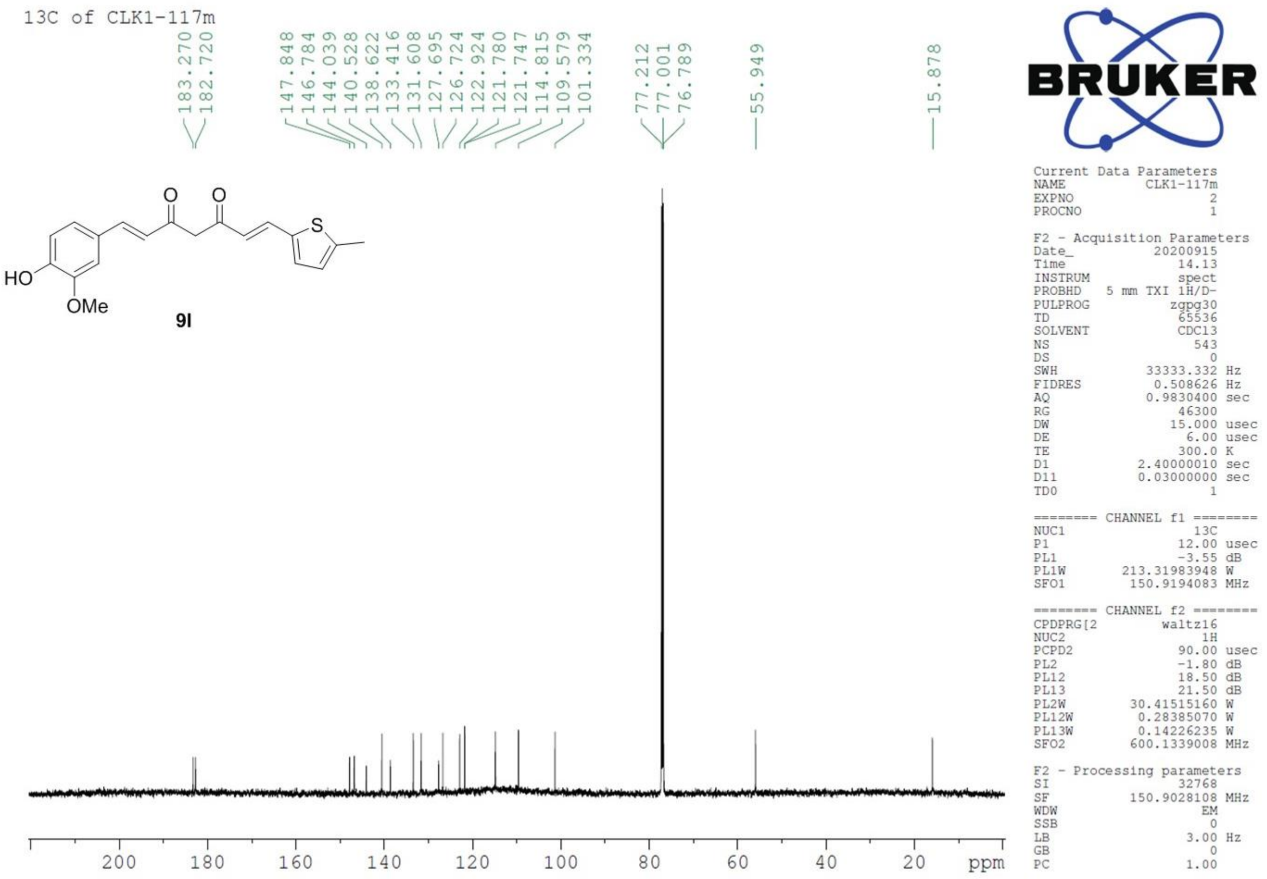

Figure S26. ^{13}C NMR (150 MHz, $CDCl_3$) for compound **9l**.

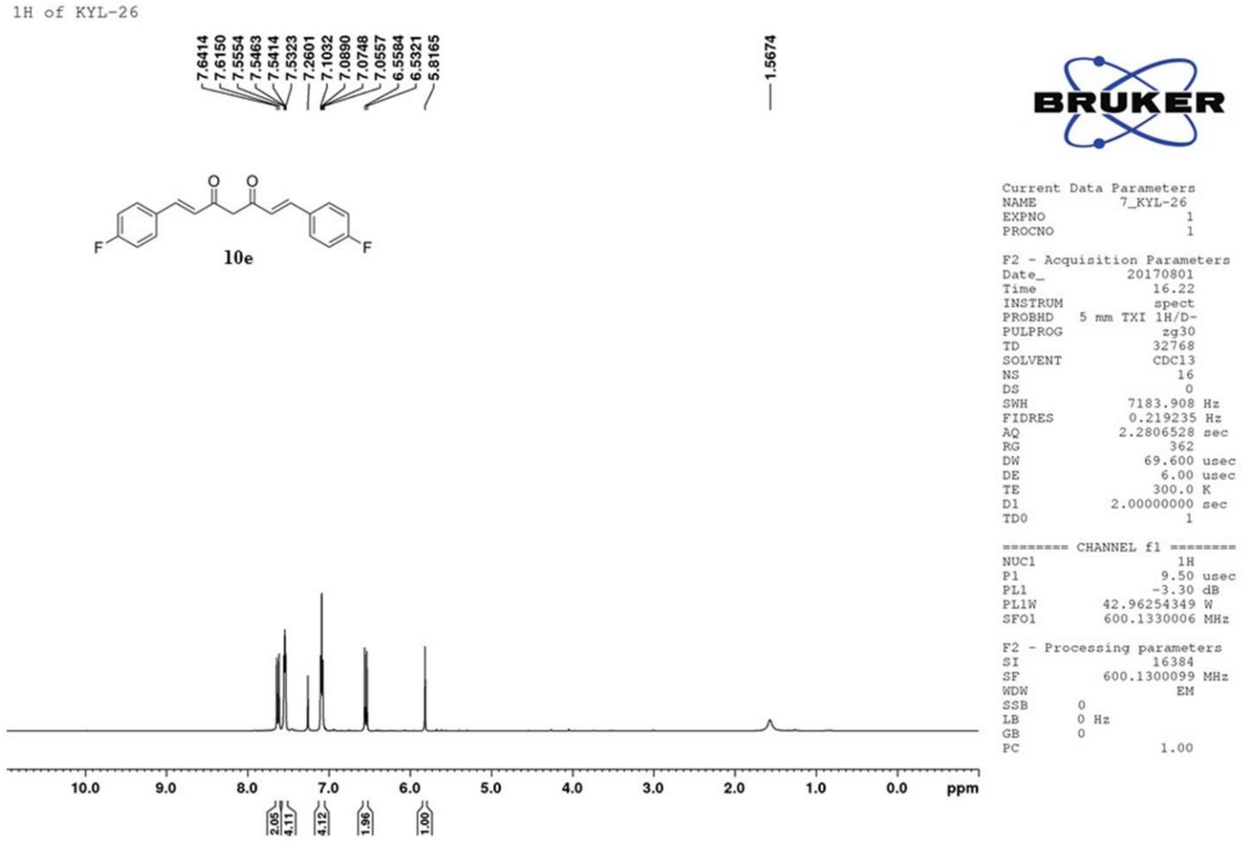

Figure S27. ^{1}H NMR (600 MHz, $CDCl_3$) for compound **10e**.

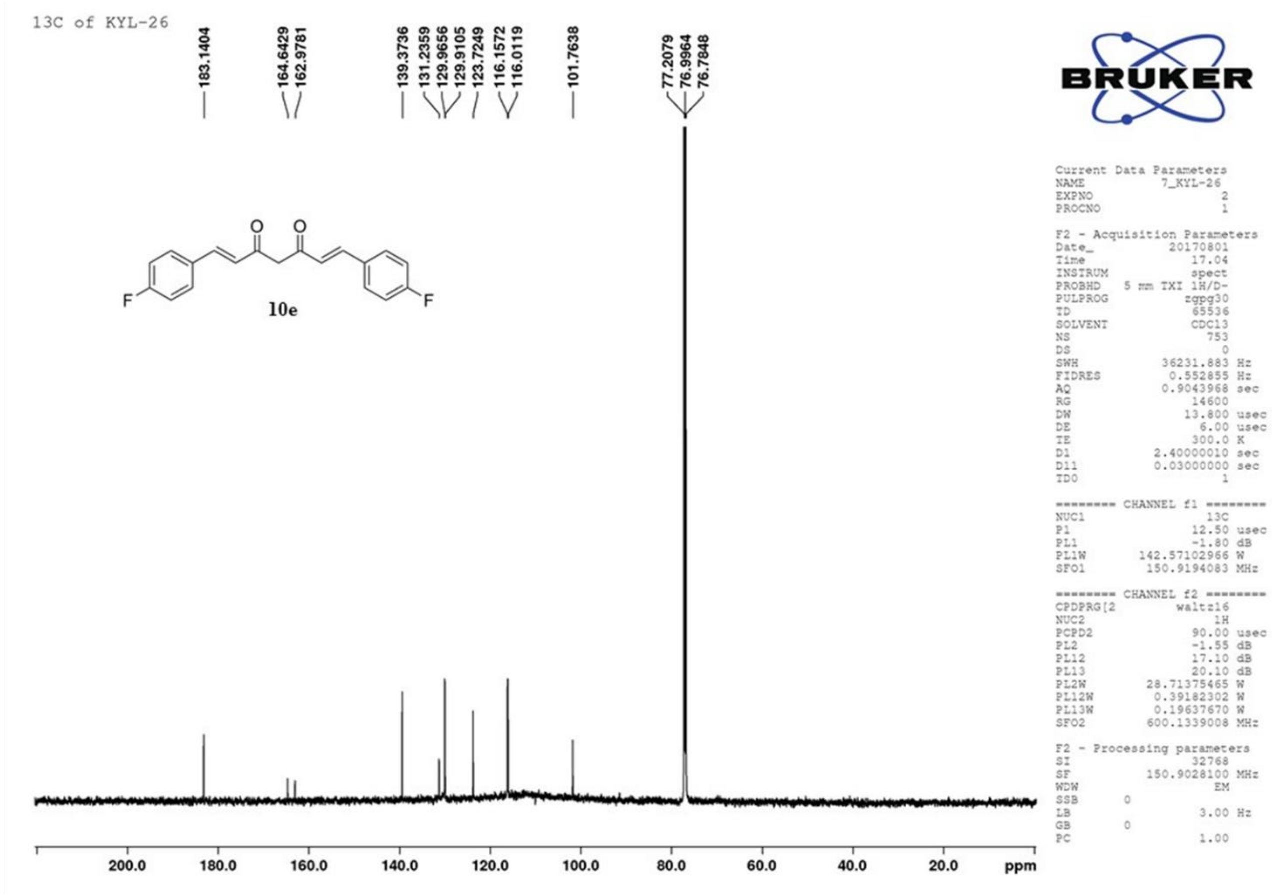

Figure S28. ^{13}C NMR (150 MHz, $CDCl_3$) for compound **10e**.

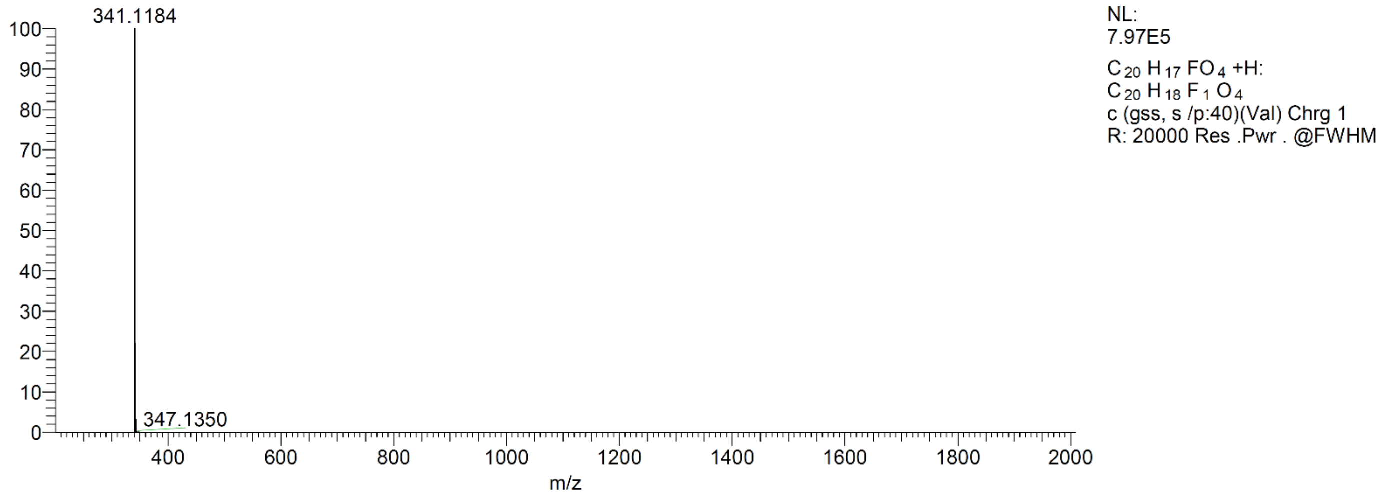

Figure S29. HR-ESI-MS for compound **9a**
